# Flaxseed Increases Animal Lifespan and Reduces Ovarian Cancer Severity by Toxically Augmenting One-Carbon Metabolism

**DOI:** 10.3390/molecules26185674

**Published:** 2021-09-18

**Authors:** William C. Weston, Karen H. Hales, Dale B. Hales

**Affiliations:** 1 Department of Molecular, Cellular & Systemic Physiology, School of Medicine, Southern Illinois University, Carbondale, IL 62901, USA; william.c.weston1@gmail.com; 2 Department of Obstetrics & Gynecology, School of Medicine, Southern Illinois University, Carbondale, IL 62901, USA; khales@siumed.edu

**Keywords:** flaxseed, nutrition, aging, lifespan, cancer, metabolism, liver, diet, ovarian, chicken

## Abstract

We used an LC-MS/MS metabolomics approach to investigate one-carbon metabolism in the plasma of flaxseed-fed White Leghorn laying hens (aged 3.5 years). In our study, dietary flaxseed (via the activity of a vitamin B6 antagonist known as “1-amino d-proline”) induced at least 15-fold elevated plasma cystathionine. Surprisingly, plasma homocysteine (Hcy) was stable in flaxseed-fed hens despite such highly elevated cystathionine. To explain stable Hcy, our data suggest accelerated Hcy remethylation via BHMT and MS-B12. Also supporting accelerated Hcy remethylation, we observed elevated S-adenosylmethionine (SAM), an elevated SAM:SAH ratio, and elevated methylthioadenosine (MTA), in flaxseed-fed hens. These results suggest that flaxseed increases SAM biosynthesis and possibly increases polyamine biosynthesis. The following endpoint phenotypes were observed in hens consuming flaxseed: decreased physiological aging, increased empirical lifespan, 9–14% reduced body mass, and improved liver function. Overall, we suggest that flaxseed can protect women from ovarian tumor metastasis by decreasing omental adiposity. We also propose that flaxseed protects cancer patients from cancer-associated cachexia by enhancing liver function.

## 1. Introduction

Our laboratory utilizes the White Leghorn laying hen as a pre-clinical animal model for the study of ovarian cancer. Laying hens spontaneously develop “biologically natural” ovarian tumors starting at around two years of age. This is the age when the hen will have ovulated as frequently as a woman who is approaching menopause [1,2,3,4,5]. As such, ovarian cancer risk is tightly correlated with the frequency of ovulation. Our lab also studies the effect of dietary flaxseed as a nutritional intervention to reduce ovarian cancer severity in laying hens. Over the past 12 years, we have illustrated that flaxseed alters the estrogenic, inflammatory, epigenetic, angiogenic, and apoptotic microenvironment of laying hen ovarian tumors [6,7,8,9,10] while also increasing the bird’s lifespan [11]. In our current paper, we are the first lab to elucidate a one-carbon metabolic phenomenon that allows flaxseed to shift cancer into a disease that animals can “survive with” instead of “die from”. This speaks highly about flaxseed’s potential as an anti-cancer food, especially considering ovarian cancer’s reputation as a highly lethal carcinoma [12].

In general, one-carbon metabolism represents the process of donating, modifying, and transferring single-carbon units within the folate cycle, the methionine (Met) cycle, the transsulfuration pathway, and phospholipid metabolism [13,14,15]. Homocysteine (Hcy), a non-protein-coding thiol amino acid, is a central molecule that connects the major pathways of one-carbon metabolism. It is important to appreciate that, in animals, Hcy is subject to four metabolic fates:

(1) Remethylation to Met via betaine homocysteine methyltransferase (BHMT) or via methionine synthase complexed with vitamin B12 (MS-B12) [16,17];
(2) Oxidation to cystathionine via cystathionine beta synthase (CBS) [18];
(3) Adenosylation to S-adenosylhomocysteine (SAH) via the bidirectional enzyme S-adenosylhomocysteine hydrolase (SAHH) [19];
(4) Accumulation (where Hcy is not remethylated, oxidized, or adenosylated), leading to a pathological condition known as hyperhomocysteinemia (HHcy) [20].

**Figure 1:**
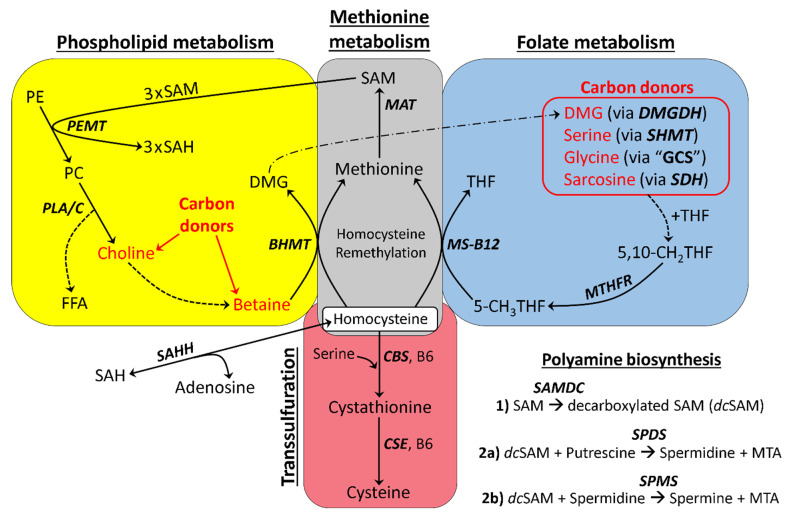
Simplified model of one-carbon metabolism: The irreversible oxidation of Hcy to cystathionine requires the B6-dependent enzyme CBS. Likewise, the oxidation of cystathionine to cysteine requires the B6-dependent enzyme CSE. The BHMT reaction utilizes betaine (which is derived from choline) as a methyl group donor, while MS-B12 utilizes 5-CH_3_THF as a methyl group donor. Specific molecules (e.g., DMG, serine, glycine, and sarcosine) act as carbon donors in the presence of THF to form 5,10-CH_2_THF in the folate cycle. 5,10-CH_2_THF contributes directly or indirectly (depending on mitochondrial or cytosolic localization) to the formation of 5-CH_3_THF, via MTHFR. After Hcy is remethylated via BHMT or MS-B12, the newly formed Met can be adenosylated via MAT to form SAM. Three molecules of SAM are consumed via the PEMT reaction, which produces one molecule of PC and three molecules of SAH. SAH is then hydrolyzed via the bidirectional enzyme SAHH, to yield adenosine and Hcy. BHMT = betaine homocysteine methyltransferase; CBS = cystathionine beta synthase; CSE = cystathionase; *dc*SAM = decarboxylated SAM; DMG = dimethylglycine; FFA = free fatty acid; Hcy = homocysteine; Met = methionine; MS-B12 = methionine synthase complexed with vitamin B12; MTA = methylthioadenosine; MTHFR = methylene tetrahydrofolate reductase; PC = phosphatidylcholine; PE = phosphatidylethanolamine; PLA/C/D = phospholipase A, C, or D; SAH = S-adenosylhomocysteine; SAHH = S-adenosylhomocysteine hydrolase; SAM = S-adenosylmethionine; SAMDC = SAM decarboxylase; SPDS = spermidine synthase; SPMS = spermine synthase; THF = tetrahydrofolate.

In Figure 1, we provide a simplified model of one-carbon metabolism where we illustrate the major pathways that are integral to our research. When evaluating this model, notice the “carbon donor” molecules (highlighted in red) that feed single-carbon units into one-carbon metabolism. For an in-depth look at one-carbon metabolism, we refer the reader to our expanded model shown in Supplementary Materials, Figure S1 [13,17,21,22,23,24,25,26,27,28,29,30,31,32,33,34,35,36,37,38,39,40,41,42,43,44,45,46,47,48,49].

Nature provides a myriad of ways for animals to encounter vitamin B6 (B6) antagonizing molecules [50]. Linatine, the B6 antagonizing molecule in flaxseed, was isolated and characterized in 1967, as 1-[(*N*-γ-l-glutamyl)amino]-d-proline [51]. In 2014, researchers confirmed via UPLC-MS that linatine is conserved in *Linum usitatissimum* (common flaxseed), independent of geographic cultivar [52], meaning that linatine should be detected at bioactive levels in all varieties of flaxseed. Linatine becomes bioactivated in the animal’s stomach or gizzard when it is hydrolyzed into glutamic acid and 1-amino d-proline (1ADP). 1ADP functions as the putative B6 antagonist. 1ADP forms an inert hydrazone with pyridoxal phosphate (PLP), thereby reducing the physiological B6 level [51]. The earliest record of flaxseed’s anti-B6 effect came in 1928, when researchers observed reduced growth in flaxseed-fed chicks [53]. Twenty years later, researchers discovered that B6 supplementation reverses flaxseed’s antigrowth effects in chicks [54] and turkey poults [55], suggesting that Galliforme birds (in general) are sensitive to 1ADP. More recently, flaxseed extracts and synthetic 1ADP were used to reduce CBS and cystathionase (CSE) activity in mammalian liver models [56,57], indicating that 1ADP’s biggest effect is hepatic. Insufficient physiological B6 levels are commonly accompanied by elevated plasma cystathionine [40,58], because cystathionine gets trapped in transsulfuration when it cannot react through the B6-dependent enzyme CSE [59,60].

Our research spotlights a novel nutritional synergy between flaxseed and avian metabolism. Specifically, we show that flaxseed perturbs transsulfuration flux in the laying hen without inducing HHcy. Instead of experiencing HHcy, our data suggest that flaxseed-fed hens exhibit accelerated Hcy remethylation via BHMT and MS-B12. This rerouting of one-carbon metabolism leads to increased SAM synthesis, an increased SAM:SAH ratio, and possibly increased polyamine biosynthesis. The associated biological outcomes include decreased physiological aging, increased empirical lifespan, 9–14% reduced body mass, and improved liver function. These phenotypes reflect flaxseed’s life extending properties as well as flaxseed’s cancer preventative properties. We describe the mechanism through which flaxseed might act via one-carbon metabolism to protect women from ovarian tumor metastasis, and we discuss how flaxseed modifies one-carbon metabolism to protect patients from cancer-associated cachexia.

## 2. Results

### 2.1. Heatmap and PLSDA Analysis of Plasma Metabolites

A targeted LC-MS/MS metabolomics approach was used to measure 108 metabolites in the plasma of 3.5-year-old White Leghorn laying hens that consumed one of six isocaloric diets for 325 days. The diets that we used are abbreviated in our study as follows: control diet (CTL), 10% defatted flaxseed meal (DFM), 15% whole flaxseed (WFX), 5% flaxseed oil (FXO), 5% corn oil (CRN), and 5% fish oil (FSH). The DFM diet was created via a cold-press fat extraction method that left approximately 40% residual fat in the final product (data not shown). As such, the DFM diet (being 10% DFM) had approximately 26.7% of the fat content of the WFX diet. In-depth formulations of our diets can be seen in Section 5 (Materials and Methods).

**Figure 2:**
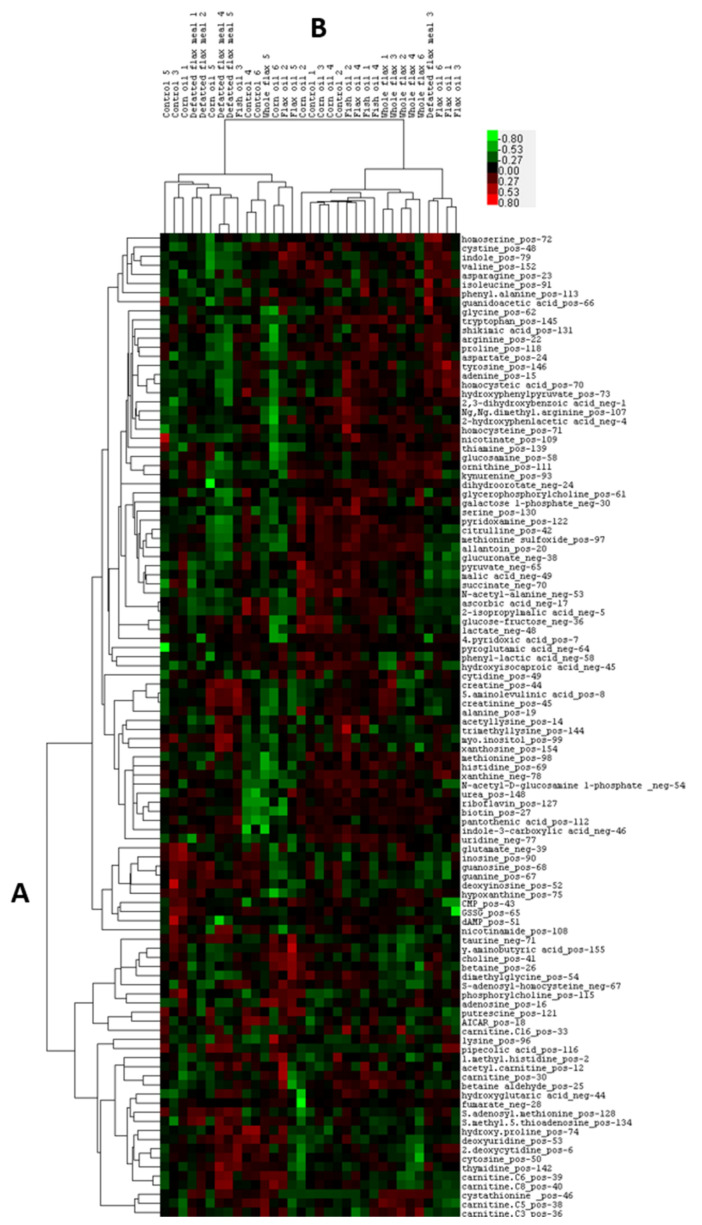
Heatmap illustrating grouped hierarchies of metabolites by diet group: Metabolites (*n* = 108 metabolites) from hen plasma were analyzed via LC-MS/MS and organized based on the clustering of their VIP scores across diet. In dendrogram (**A**), metabolites are organized according to their similarity across diet samples. In dendrogram (**B**), diet samples are organized according to their similarity across metabolites. Green illustrates below average standard deviation, and red illustrates above average standard deviation.

All LC-MS/MS peak values for metabolites were converted to variable importance in projection (VIP) scores prior to analysis. These VIP scores were visualized globally via two-dimensional heatmapping (Figure 2) and via a partial least squares discriminant analysis (PLSDA) (Supplementary Materials, Figure S2). The PLSDA informed us that the DFM hens displayed the most discrete metabolite cluster. The PLSDA also indicated that the WFX hens shared a clustered zone that overlapped with FXO hens and somewhat with DFM hens, suggesting a common overlap between the oil and non-oil components of flaxseed. The CTL hens and CRN hens appeared to be more widely dispersed, while the FSH hens expressed a unique cluster.

### 2.2. Perturbed B6 Levels Concomitant with Elevated Cystathionine

**Figure 3:**
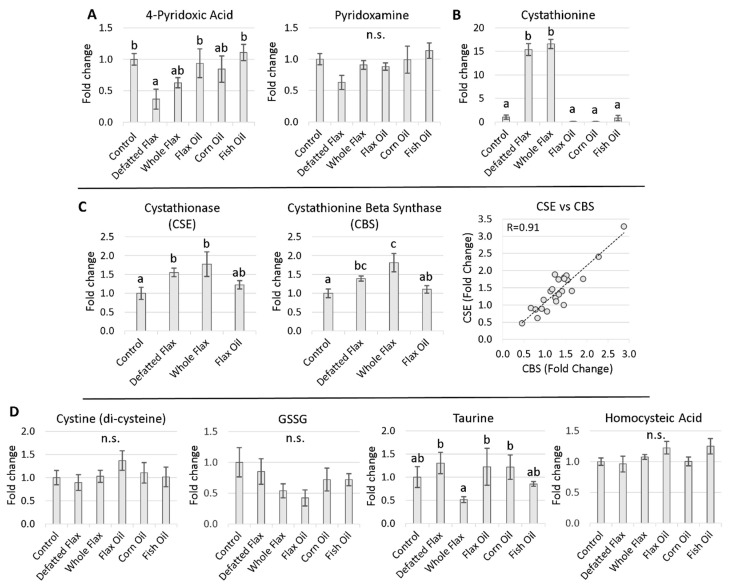
Estimates of vitamin B6 metabolism, transsulfuration, and sulfur-based metabolites generated downstream of transsulfuration: Hen plasma samples were measured via LC-MS/MS. Plasma markers for B6 metabolism were measured (**A**). Plasma markers for transsulfuration flux were also measured (**B**). Gene transcripts for CBS and CSE were measured in hen liver homogenates via qPCR (using GAPDH as reference) (**C**). Thiol metabolites that were generated subsequent to transsulfuration were measured (**D**). VIP scores of plasma metabolites and fold changes of gene transcripts were analyzed via one-way ANOVA (Duncan’s post-test, *p* < 0.05). Groups lacking a similar letter (i.e., a,b,c) are significantly different. “n.s.” is used to illustrate a non-significant ANOVA. A sample size of 6 was used for each group in (**C**). The sample sizes for the groups in (**A**,**B**,**D**), are stated in the Materials and Methods section (Section 5.3). The F-test result for 4PA was *p* < 0.056. Error bars are +/− SEM.

Vitamin B6 metabolism was perturbed by the two diets that contained linatine (i.e., DFM and WFX). For example, 4PA was significantly reduced by DFM and almost (*p* < 0.17) reduced by WFX (Figure 3A). 4PA is the carboxylated end-product of the B6 vitamer cycle. Similar to 4PA, the level of pyridoxamine was, on average, lowest in the DFM hens. For 4PA and pyridoxamine, the greatest mean differences occurred between DFM hens and FSH hens, suggesting similar regulation of B6 (Figure 3A). Next, transsulfuration flux was extensively perturbed according to 15.4-fold and 16.6-fold elevated cystathionine in DFM hens and WFX hens, respectively (Figure 3B). The genes for CSE and CBS showed approximately 1.5-fold to 1.75-fold increased expression in the livers of hens consuming either DFM or WFX (Figure 3C), possibly indicating compensation to help overcome reduced transsulfuration flux. We did not assay CSE or CBS gene expression in CRN hens or FSH hens. Cystine (a di-cysteine molecule used for transmembrane passage of cysteine) was not affected by diet. Among other thiol metabolites, taurine was slightly decreased by WFX, while diet did not affect GSSG (i.e., reduced glutathione) or homocysteic acid (Figure 3D). We did not have GSH (i.e., oxidized glutathione) in our metabolomics data-set. Urea, creatinine, and the urea:creatinine ratio, were not affected by diet (Supplementary Materials, Figure S3), providing evidence that renal filtration was stable across diets.

### 2.3. Methionine Cycle Metabolites

**Figure 4:**
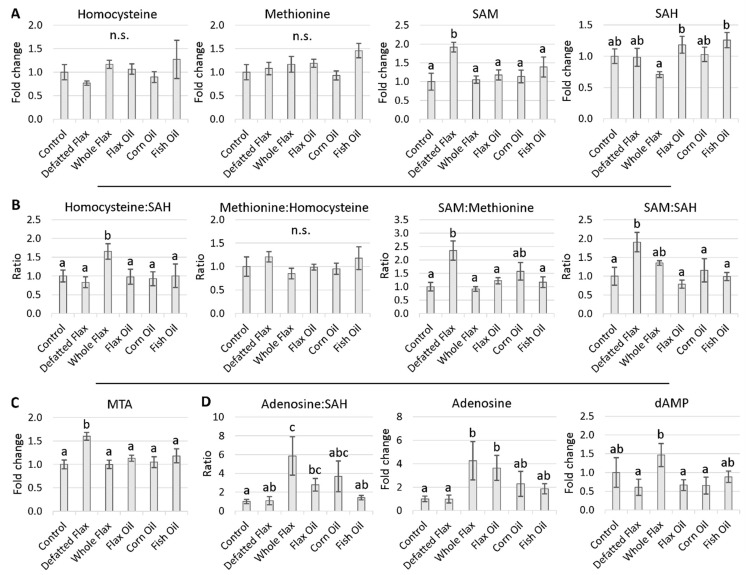
Plasma estimate of methionine cycle metabolites and associated metabolites: Hen plasma samples were measured via LC-MS/MS. VIP scores of metabolites were analyzed via one-way ANOVA (Duncan’s post-test, *p* < 0.05). Groups lacking a similar letter (i.e., a,b,c) are significantly different. “n.s.” is used to illustrate a non-significant ANOVA. The four methionine cycle metabolites (**A**) as well as their ratios (**B**) are depicted. Plasma MTA was estimated (**C**), and we have multiple proxy markers to estimate SAHH activity (**D**). The sample size from each group is listed in the Materials and Methods section (Section 5.3). The F-test result for Homocysteine:SAH was *p* < 0.066. Error bars are +/− SEM.

Mammalian models suggest that plasma Hcy should be elevated when cystathionine is as little as 4-fold elevated [56]. Interestingly, we observed non-elevated Hcy (Figure 4A) despite cystathionine being greater than 15-fold elevated (Figure 3B). These results indicate that the remethylation pathway via BHMT and MS-B12 was accelerated in flaxseed-fed hens. We received support for this notion when we observed that SAM and the SAM:SAH ratio were both elevated 1.9-fold in DFM hens (Figure 4A,B). This is the first indication that flaxseed’s anti-B6 effect associates with elevated SAM synthesis in animals. The SAM:Met ratio was high (i.e., 2.35-fold elevated) in DFM hens, consistent with increased methionine adenosyltransferase (MAT) activity (Figure 4B). Methylthioadenosine (MTA) was also elevated in DFM hens 1.6-fold (Figure 4C). MTA is a SAM-derived molecule that is produced when decarboxylated SAM reacts through polyamine biosynthesis [61] (see Figure 1 and Supplementary Materials, Figure S1). The Pearson coefficient between SAM and MTA was R = 0.66 across 33 samples, indicating a strong relationship.

SAM was not elevated in WFX hens (unlike in DFM hens) (Figure 4A). We suspect that elevated activity from phosphatidylethanolamine methyltransferase (PEMT) might be why SAM was stable in WFX hens. PEMT is arguably the greatest SAM-consuming enzyme in biology [30,62,63]. We hypothesize that PEMT hyperactivation drove the consumption of all excess SAM in WFX hens, thus, causing SAM to appear stable. In support of PEMT hyperactivation in WFX hens, we observed that the Hcy:SAH ratio and the adenosine:SAH ratio were simultaneously elevated (Figure 4B,D), while SAH was slightly decreased (Figure 4A). This provides an estimate of increased SAHH activity, because Hcy and adenosine are products of the SAHH reaction, while SAH is substrate. It is likely that SAHH activity would be elevated in WFX hens, because PEMT generates a large volume of SAH [30,63,64]. SAH inhibits methyltransferases such as PEMT [30,36]; therefore, SAHH hyperactivation would complement PEMT hyperactivation. Adenosine was elevated 4-fold on average in WFX hens, while dAMP (a molecule that is readily synthesized from adenosine) was slightly elevated in WFX hens (Figure 4D). These elevations provide further evidence that SAHH activity might be increased.

Another major consumer of SAM is guanidinoacetate methyltransferase (GAMT) [65]. GAMT is the enzyme that is responsible for creatine synthesis. The metabolomics data indicate that flaxseed had no effect on creatine levels or GAMT activity, according to plasma estimates of creatine, guanidinoacetic acid, or the ratio of the two (Supplementary Materials, Figure S4), informing us that PEMT (and not GAMT) was more likely to have accounted for excess SAM consumption in WFX hens. Importantly, other researchers have noted that flaxseed-fed hens have a high hepatic PC:PE ratio of 3.78 [66], possibly indicating elevated PEMT activity and, thus, elevated SAM consumption [63,67,68].

### 2.4. Evidence of Accelerated Hcy Remethylation

#### 2.4.1. One-Carbon Donors That Fuel BHMT: Choline and Betaine

**Figure 5:**
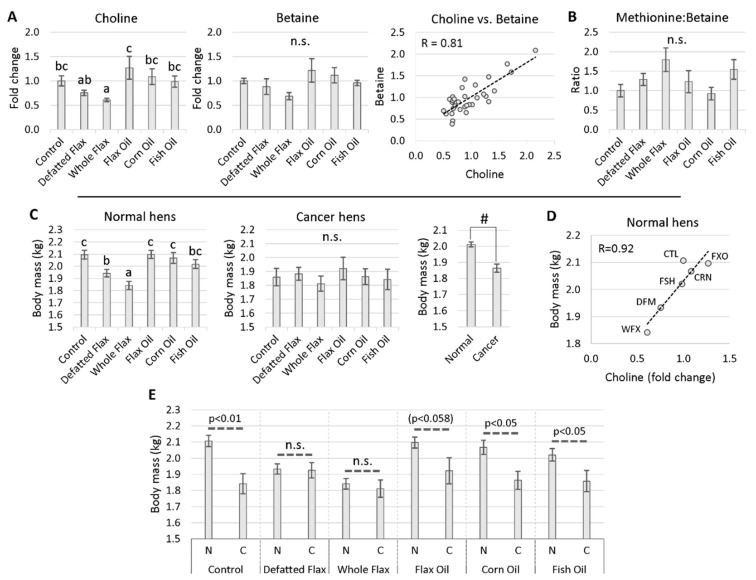
One-carbon donor molecules that fuel BHMT, in association with hen body mass: Hen plasma samples were measured via LC-MS/MS. VIP scores of metabolites were analyzed via one-way ANOVA (Duncan’s post-test, *p* < 0.05). Groups lacking a similar letter (i.e., a,b,c) are significantly different. “n.s.” is used to illustrate a non-significant ANOVA or T-test. Choline and betaine were evaluated in plasma (**A**), and the Met:betaine ratio was observed (**B**). We tested the effect of diet on body mass, using one-way ANOVA (Duncan’s post-test, *p* < 0.05) (**C**). Body mass was also contrasted between normal hens and cancerous hens, using the Student’s *t*-test for unequal variance (two-tailed; “**#**” indicates *p* < 0.01) (**C**). The Pearson coefficient between mean body mass and mean choline content was measured (**D**). In (**E**), the body masses between cancerous hens (“C”) and normal hens (“N”) were compared within diets, using the Student’s *t*-test for unequal variance. The sample sizes for groups in (**E**) are, by order of appearance: 86, 31, 85, 23, 73, 22, 84, 22, 62, 29, 70, and 24. The sample size from each group in (**A**) is listed in the Materials and Methods section (Section 5.3). The sample size from each group in (**C**) is listed in the Materials and Methods section (Section 5.2). One outlier was removed from the whole flax diet group in the graph of choline (**A**). Error bars are +/− SEM.

To evaluate the hypothesis that BHMT activity was elevated in flaxseed-fed hens, we measured the carbon donors that fuel the BHMT reaction. Specifically, we evaluated the levels of choline and betaine. In WFX hens, plasma choline was exceptionally low, ranging from 39–52% lower than all other hens except versus DFM hens (Figure 5A). In DFM hens, plasma choline was only lower than versus FXO hens (Figure 5A). These results mean that choline was decreased by the linatine-containing diets (i.e., DFM or WFX). Betaine, the methyl group donor for BHMT was, on average, lower in WFX hens than versus FXO hens (Figure 5A). A high Pearson correlation coefficient between choline and betaine suggested an oxidative vector between the two molecules (Figure 5A). This vector should exist, because choline is oxidized for the purpose of replenishing betaine, especially in the liver. Choline’s role as a betaine replenisher could be a key reason why betaine was stable in the ANOVA. Another key factor contributing to betaine’s stability could be the hepatocyte’s specialized ability to store high concentrations of betaine. Unlike betaine, hepatocytes do not specialize in storing soluble choline, making choline easily depletable. Choline is stored primarily in the form of phosphatidylcholine (PC) in the plasma membrane. The Met:betaine ratio (a proxy for BHMT activity) was, on average, highest in WFX hens (Figure 5B), suggesting that BHMT activity might be elevated in WFX hens.

The plasma levels of BHMT’s negative regulators (i.e., DMG, SAM, and MTA) favored net gain of BHMT function in WFX hens (Supplementary Materials, Table S1). In other words, the “BHMT feedback gate” was wide open for WFX hens. DFM had a different level of BHMT’s negative regulators, such that the net effect on BHMT function might represent little to no net change (Supplementary Materials, Table S1). Our data suggest also that the FXO diet increases BHMT mRNA levels in the hen’s liver (Supplementary Materials, Figure S5).

#### 2.4.2. Hen Body Mass Was Reduced in Association with Choline Content

For “Normal hens” (i.e., non-cancerous hens) we observed a 14% reduced body mass in WFX hens versus CTL hens, and we observed a 9% reduced body mass in DFM hens versus CTL hens (Figure 5C). Diet had no effect on hen body mass in “Cancer hens”. The wasting effect of cachexia was obvious in cancerous hens, because cancerous hens weighed approximately 7% less than normal hens on average (Figure 5C, “#”). The Pearson correlation between mean choline level and mean body mass was high across diet groups in “Normal hens” (Figure 5D), suggesting a strong relationship between choline metabolism and body mass.

The body masses of normal hens and cancerous hens were similar within the DFM diet and WFX diet (Figure 5E). In contrast, the body masses of hens in CTL, FXO, CRN, and FSH, were significantly lower in the presence of cancer (Figure 5E). These cancer-dependent reductions in body mass likely indicate the wasting effects of cachexia.

#### 2.4.3. One-Carbon Donors That Fuel the Folate Cycle (i.e., Fuel 5,10-CH_2_THF Synthesis)

**Figure 6:**
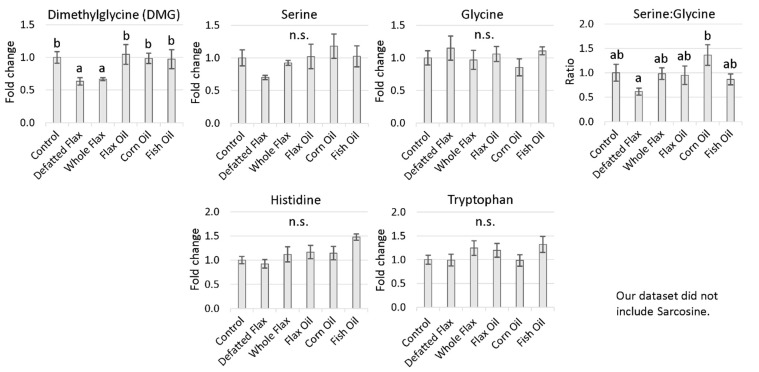
Folate cycle carbon donors that contribute to 5,10-CH_2_THF synthesis: Hen plasma samples were measured via LC-MS/MS. VIP scores of metabolites were analyzed via one-way ANOVA (Duncan’s post-test, *p* < 0.05). Groups lacking a similar letter (i.e., a,b) are significantly different. “n.s.” is used to illustrate a non-significant ANOVA. The sample size from each diet group is listed in the Materials and Methods section (Section 5.3). Error bars are +/− SEM.

We suspect elevated MS-B12 activity; therefore, we wanted to evaluate carbon donors that are consumed during the synthesis of 5,10-CH_2_THF. To do this, we measured the plasma levels of DMG, serine, glycine, histidine, and tryptophan (Figure 6). DMG was decreased 33% and 37%, respectively, in WFX hens and DFM hens. This is meaningful, because the only enzyme known to catabolize DMG is dimethylglycine dehydrogenase (DMGDH), located in the folate cycle [42]. DMGDH catabolizes DMG alongside with THF to yield sarcosine and 5,10-CH_2_THF. The decreased DMG level in DFM hens and WFX hens suggests that DMGDH activity and 5,10-CH_2_THF synthesis might be elevated in those animals. Elevated 5,10-CH_2_THF synthesis should help to accelerate Hcy remethylation through MS-B12. We also observed that serine was, on average, lowest in DFM hens (especially versus CRN hens), and glycine was, on average, highest in DFM hens (especially versus CRN hens) (Figure 6). We uncovered an important signal when we measured the serine:glycine ratio. The serine:glycine ratio was decreased in DFM hens versus CRN hens, possibly indicating increased conversion of serine to glycine through serine hydroxymethyltransferase (SHMT) (Figure 6). Under normal circumstances, serine is the primary carbon donor toward the synthesis of 5,10-CH_2_THF, through SHMT [14]. SHMT and DMGDH provide two critical entry points for carbon into the folate cycle. Histidine and tryptophan (two molecules that minorly contribute to 5,10-CH_2_THF synthesis) were stable across diets, although histidine was, on average, lowest in DFM hens (Figure 6).

We also conducted k-means clustering analysis to visualize how diet groups clustered according to one-carbon donor molecules (Supplementary Materials, Figure S6). According to k-means clustering, DFM hens exhibited the tightest and most unique regulation of one-carbon donor molecules, while WFX hens shared a noticeable overlap with both DFM hens and FXO hens. Interestingly, the k-means clustering patterns for DFM hens, WFX hens, and FXO hens paralleled the patterns that we observed during the PLSDA analysis (Supplementary Materials, Figure S2). Lastly, we measured the mRNA levels of methionine synthase (MS) and methylene tetrahydrofolate reductase (MTHFR), in liver homogenates, and neither gene appeared to be regulated by diet (Supplementary Materials, Figure S5).

### 2.5. Survival and Physiological Aging of Hens

**Figure 7:**
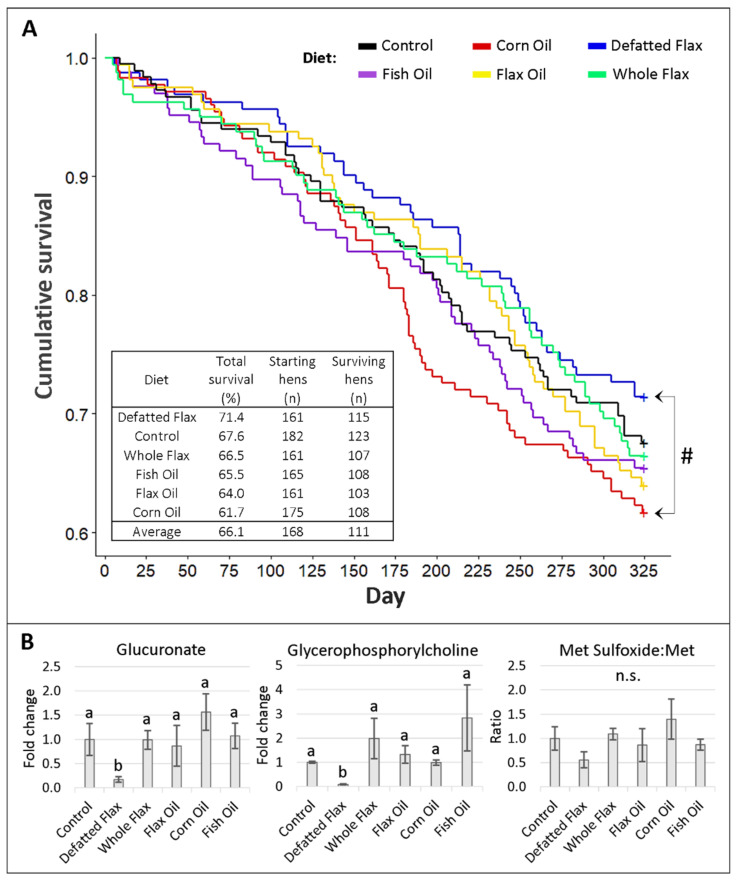
Kaplan–Meier survival analysis and physiological biomarkers of aging: Kaplan–Meier analysis was conducted across diet groups from day 1 to day 325 (**A**). The “#” indicates a reduced Cox proportional hazard comparing DFM hens to CRN hens (hazard exp{coef} = 0.685; *p* < 0.05). In addition, there are shown three biomarkers of aging, measured via LC-MS/MS (**B**). VIP scores of metabolites were analyzed via one-way ANOVA (Duncan’s post-test, *p* < 0.05). Groups lacking a similar letter (i.e., a,b) are significantly different. “n.s.” is used to illustrate a non-significant ANOVA. For glycerophosphorylcholine, one outlier was removed from Control, Defatted Flax, and Corn Oil. Error bars are +/− SEM.

71.4% of the hens from the DFM diet group survived the study, representing the best overall survival rate. The lowest survival rate came from CRN hens with a 61.7% survival rate. CTL hens and WFX hens displayed above average survival, while FSH hens and FXO hens displayed below average survival (Figure 7A). The Kaplan–Meier survival curve indicated that DFM hens maintained the best cumulative survival except for a few brief moments (Figure 7A). Notably, DFM hens were the only animals to maintain better survival than CTL hens from day 30 until the study’s completion. We also detected a reduced Cox hazard in DFM hens than versus CRN hens (denoted by “#” in Figure 7A; also see Supplementary Materials, Table S2).

To estimate physiological aging, we evaluated three distinct biomarkers that have been previously used to elucidate aging in human and non-human studies [69,70,71]. Glucuronate, a molecule involved in urinary waste excretion, was widely lower in DFM hens versus all other diet groups, with the biggest mean difference occurring between DFM hens and CRN hens (Figure 7B). This was interesting because the Cox proportional hazard was also decreased in DFM hens versus CRN hens. Glycerophosphorylcholine (GPC), a well-known plasma osmolyte, was lower in DFM hens versus all other diet groups (Figure 7B). Lastly, the Met sulfoxide:Met ratio (a marker for systemic oxidative stress) was, on average, lowest in DFM hens (especially versus CRN hens) (Figure 7B). Altogether, we have a mathematical signal (i.e., Kaplan–Meier curve plus Cox hazard analysis) and a physiological signal (i.e., plasma biomarkers) to compositely suggest increased lifespan and decreased aging in DFM hens.

### 2.6. Flaxseed’s Effect on Microarray Feature Expression in the Hen Ovary

#### 2.6.1. Flaxseed Downregulates Feature Expression in the Ovary

After observing an increased SAM:SAH ratio and an increased MTA level in flaxseed-fed hens (Figure 3B,D), we hypothesized that flaxseed could regulate molecular processes in tissues that are distal from the liver. To challenge this, we reanalyzed a microarray data set that measured 44,000 mRNA features in normal and cancerous ovaries of hens consuming a 10% whole flaxseed diet (WFX10) or a control diet (CTL). The original analysis [9] implemented *t*-tests that compared feature expression between the two diets in normal and cancerous tissues. Our reanalysis provides a summary of those *t*-tests according to three strata of statistical significance (i.e., *p* < 0.10, *p* < 0.05, *p* < 0.01) (see Table 1).

The WFX10 diet primarily downregulated microarray feature expression in normal and cancerous ovaries, across all three strata of statistical significance (Table 1). This was evidenced by the number of downregulated features as well as the ratio of “downregulated-to-upregulated” features. This ratio was high, reaching as much as 4.38 in normal ovaries and 3.24 in cancerous ovaries (Table 1). Clearly, the trend is toward “downregulation of the transcriptome” in the ovaries of WFX10-fed hens, independent of tissue disease state (i.e., normal or cancer). The strength of effect is more pronounced in normal ovaries; however, it is noteworthy that WFX10 imparts this effect during cancer.

#### 2.6.2. Flaxseed Downregulates Features for SAM-Dependent Methyltransferases

In normal hen ovaries, 156 features were affected by diet at the *p* < 0.01 level of significance (Table 1). Only 40 of these 156 features contained identifiable information. Of these 40 identifiable features, we observed four SAM-dependent methyltransferases, one demethylase, and one protein related to cobalamin metabolism (Table 2). In other words, 15% of the 40 identifiable features were related to SAM-dependent metabolism. Each of these six features was downregulated between −1.67 and −2.10-fold, suggesting that flaxseed downregulates SAM-dependent metabolism transcriptionally in the hen ovary. If we stretch the *p*-value threshold to *p* < 0.02, the WFX10 diet also downregulated the expression of DNA Methyltransferase 2 (DNMT2) and RNA guanine-N7 methyltransferase (RNMT) by −1.76-fold each in normal ovaries (data not shown). Altogether, our microarray data indicate that flaxseed might downregulate the transcription of SAM-dependent methyltransferases, particularly methyltransferases that methylate histone proteins, mRNA, tRNA, rRNA, and DNA. This is a signal that flaxseed might downregulate the “methyltransferome” of the hen ovary, and this could very well mediate an anti-aging effect. These results, having occurred in flaxseed-fed hens, are likely associated with an elevated plasma SAM:SAH ratio and/or elevated plasma MTA level.

Interestingly, we identified that CBWD1 (cobalamin synthetase W domain-containing protein) was downregulated in the ovaries of WFX10 hens at *p* < 0.01 (Table 2). If CBWD1 influences cobalamin metabolism in chickens, then the microarray further suggests that flaxseed might decelerate MS-B12 activity in the hen’s ovary. Cobalamin (i.e., B12) is necessary for MS-B12’s methyltransferase activity between 5-CH_3_THF and Hcy. Therefore, flaxseed (by downregulating CBWD1) might decelerate MS-B12-dependent remethylation of Hcy, in the ovary. If a tissue has access to excess SAM from the plasma (i.e., as seen in flaxseed-fed hens), then the SAM biosynthetic requirement of that tissue should theoretically decline. This might explain lower levels of CBWD1 in the ovary of WFX10 hens.

#### 2.6.3. Flaxseed Increases SLC25A26 Expression and Might Reduce Ovarian Tumor Aging

In cancerous hen ovaries, 157 microarray features were affected by diet at the *p* < 0.01 level of significance (Table 1). 45 of these 157 features contained identifiable names. Among these 45 identifiable features, we observed only one feature related to SAM-dependent metabolism. Specifically, we observed a 2.07-fold upregulation of SLC25A26, which is a mitochondrial SAM carrier protein (SAMC) that transports SAM across mitochondrial membranes (Table 2). In addition, among the 45 features at *p* < 0.01, we detected a 1.88-fold increased expression of an aging-associated biomarker known as Klotho (data not shown). Elevated Klotho expression has been observed as a marker of decreased aging in studies of renal function [72,73]. Increased Klotho expression has also been associated with beneficial [74] and detrimental [75] outcomes in ovarian cancer patients.

## 3. Discussion

### 3.1. Basic Model of One-Carbon Metabolism in Flaxseed-Fed Hens

**Figure 8:**
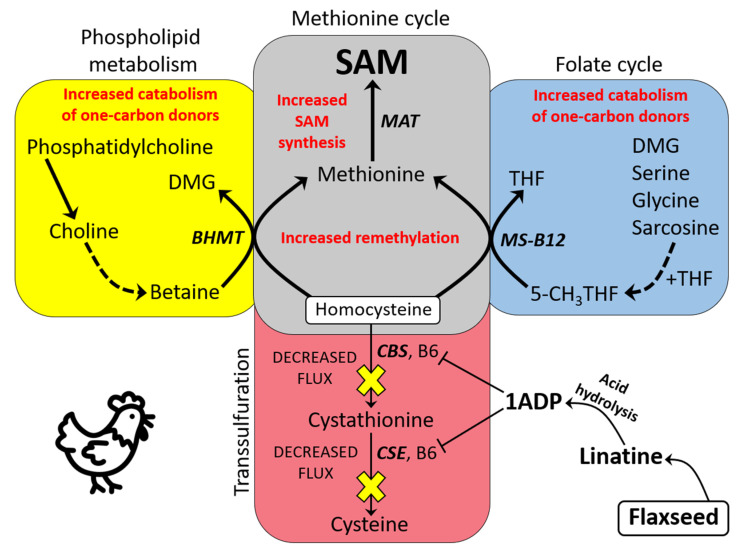
Basic model illustrating how flaxseed increases SAM synthesis in laying hens (predominantly a hepatic model). The initial effect is instigated when 1ADP (via linatine) antagonizes vitamin B6 and reduces CBS and CSE activity. In turn, this reduces carbon flux through transsulfuration and causes cystathionine trapping. Instead of developing HHcy, these animals display increased Hcy remethylation (via BHMT and MS-B12). BHMT hyperactivation induces elevated oxidation of choline and betaine, and MS-B12 hyperactivation induces elevated oxidation of molecules such as DMG and serine. The excess Met produced by the hyperactivation of BHMT and MS-B12 is then adenosylated via MAT to form an excess supply of SAM. In this manner, the SAM level and the SAM:SAH ratio of the hen will rise. 1ADP = 1-amino d-proline; 5-CH_3_THF = 5-methyl tetrahydrofolate; BHMT = betaine homocysteine methyltransferase; CBS = cystathionine beta synthase; CSE = cystathionase; DMG = dimethylglycine; Hcy = homocysteine; HHcy = hyperhomo-cysteinemia; MAT = methionine adenosyltransferase; Met = methionine; MS-B12 = methionine synthase B12; SAM = S-adenosylmethionine; SAH = S-adenosylhomocysteine; THF = tetrahydrofolate.

In this paper, we report on a novel phenomenon whereby flaxseed severely perturbs transsulfuration flux and redirects Hcy toward the Met cycle (via the suspected elevated activity of BHMT and MS-B12). The culminating effect is elevated SAM synthesis, an elevated SAM:SAH ratio, and possibly elevated polyamine biosynthesis. A basic model illustrating flaxseed’s effect on one-carbon metabolism is shown in Figure 8. We provide this basic model as a template for understanding a more complex metabolic network that we display at the end of our discussion.

### 3.2. Flaxseed Perturbs B6 Metabolism and Transsulfuration Flux: The Effect of 1ADP

Our DFM diet and WFX diet were calibrated to provide a similar mass of the cotyledon component of flaxseed (where linatine is primarily stored). The DFM diet exerted a “functionally relevant” B6 perturbation, due to its ability to concomitantly decrease 4PA and boost cystathionine. Other researchers in [56] had similar findings when B6-replete rats exhibited decreased 4PA and elevated cystathionine after consuming dietary 1ADP. There was a large effect of the WFX diet on cystathionine, regardless of the trending effect of WFX on 4PA (i.e., *p* < 0.17). In future studies, we will conduct an LC-MS/MS analysis on hen liver homogenates (instead of plasma), because liver tissue would readily capture the anti-B6 effects of flaxseed [56,76].

As far as we know, our DFM hens and WFX hens displayed the highest plasma cystathionine level ever recorded in wild-type animals consuming a B6-replete diet. These cystathionine elevations highlight the profound anti-transsulfuration effect of flaxseed in birds (in particular, Galliforme birds). We also detected increased CBS and CSE mRNA expression in the livers of DFM hens and WFX hens. This was presumably a transcriptionally regulated means to overcome reduced transsulfuration flux. Other researchers have observed increased CBS and CSE mRNA expression when chicks were exposed to specific chemical inhibitors of CSE [77]. Therefore, chickens (independent of age) attempt to overcome transsulfuration perturbations via the transcriptional upregulation of CBS and CSE.

### 3.3. SAM, SAM:SAH Ratio, and MTA Are All Elevated in Flaxseed-Fed Hens

Mammals commonly display HHcy in the presence of perturbed transsulfuration flux [20,56,76,78,79]. Therefore, avian species (or at least Galliforme birds) exhibit a uniquely reduced risk of HHcy. This suggests that avian species could provide an unforeseen, valuable animal model for the study of homocysteine homeostasis.

In our flaxseed-fed hens, the most probable fate of Hcy was accelerated remethylation into the Met cycle, via BHMT and MS-B12. Accelerated Hcy remethylation is parallel to the concept of “Met loading”, because both processes increase the Met level of the animal. Met loading also increases the rate of SAM synthesis [30]. Therefore, accelerated Hcy remethylation should accelerate SAM synthesis. The SAM level as well as the SAM:SAH ratio were highest in DFM hens, suggesting that a flaxseed diet with a low-to-moderate oil content (e.g., the DFM diet) induces the biggest elevation of the SAM level and SAM:SAH ratio. We did not observe elevated SAM or an elevated SAM:SAH ratio in WFX hens. We suspect that PEMT hyperactivation depleted all excess SAM in WFX hens, thereby causing SAM to appear non-elevated.

The pan-methyltransferase inhibitor, MTA, was elevated 1.6-fold in DFM hens. This elevation was possibly due to the increased flow of SAM through the polyamine biosynthetic pathway (i.e., Figure 1). Therefore, our modeling approach connects flaxseed dieting with polyamine biosynthesis, in hens. MTA is a methyltransferase inhibitor that protects cells by providing a “methylation buffer” against a high SAM:SAH ratio. Histone hypermethylation is a risk when the SAM:SAH ratio is elevated [30], and MTA protects cells from hypermethylation in this type of context by inhibiting histone protein methyltransferases [80,81]. The elevated MTA in DFM hens could, therefore, be indispensible for maintaining methylome homeostasis given a high SAM:SAH ratio.

**Figure 9:**
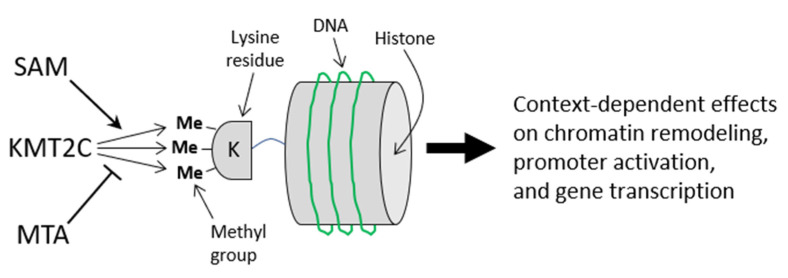
The interplay between SAM and MTA, regarding the activation of histone lysine methyltransferase enzymes such as KMT2C. Under conditions with a high SAM:SAH ratio, SAM will occupy the methyltransferase domain of KMT2C and promote methyltransferase activity toward the lysine residue (i.e., K4) on the cognate histone (i.e., H3). Under conditions with a high MTA concentration, MTA will inhibit the methyltransferase domain of KMT2C and reduce the methylation of H3K4. In this example, the interplay between SAM and MTA influences the degree to which H3K4 is methylated. Ultimately, the degree of H3K4 methylation will have a large influence on chromatin remodeling. The architecture of the chromatin will then inform how transcription factors, enhancers, and coactivators can bind to DNA consensus sequences and to nearby proteins. In turn, this will guide gene promoter activation and gene transcription.

It is likely that an interplay between the SAM:SAH ratio and the MTA level determines a “True Methylation Index.” This interplay would augment methyltransferase activity of the cell. We provide an illustration of this concept in Figure 9.

### 3.4. Increased Lifespan and Reduced Aging Associated with Elevated SAM and Elevated MTA

In our present study, the DFM hens displayed the highest cumulative survival with 71.4% survival. We previously reported 72.1% survival in 2.5-year-old hens that consumed WFX10 for one year [11]. These near-identical survival outcomes suggest that hen lifespan is similarly improved by either 10% defatted flaxseed or 10% whole flaxseed.

Flaxseed (by increasing the SAM:SAH ratio) plays a distinct role in extending animal lifespan. The SAM:SAH ratio is important for driving polyamine biosynthesis, because SAM (via decarboxylated SAM) functions as the amine contributor during the synthesis of polyamines (see Figure 1). MTA is a direct byproduct of polyamine biosynthesis, making MTA a surrogate marker for polyamine biosynthesis. Polyamines are generally associated with increased organism lifespan [82]. For example, in 24 week old mice the dietary administration of polyamines increased mouse lifespan by the 88th week of life [83].

Our research suggests that flaxseed increases lifespan in “already aged” poultry, possibly by increasing polyamine biosynthesis (according to elevated MTA). This is novel because polyamine biosynthesis tends to decline with the age of the organism or tissue [84,85,86,87]. Flaxseed might overcome this age-related decline in polyamine biosynthesis, at least in poultry. Future studies should measure spermidine and spermine production (i.e., the actual polyamines) in flaxseed-fed hens, because we were unable to evaluate their levels in this current study.

We observed at least three plasma biomarkers that were consistent with reduced aging in DFM hens. Lower levels of glucuronic acid (i.e., protonated glucuronate) indicate decreased physiological aging in mice and humans [69], and now we can add chickens to that list of animals. Of special importance, the mean differences for glucuronate matched the reduced Cox hazard between DFM hens and CRN hens. This helps to validate glucuronate as a marker for aging in poultry. Plasma GPC was previously found to be lower in “centenarian” humans versus humans who are in their 60s and 70s [70]. This means that DFM hens, by exhibiting lower plasma GPC, exhibited “healthy aging.” Clinicians have observed direct correlation between systemic oxidative stress and the Met sulfoxide:Met ratio [71]. Lastly, we suspect that a reduced plasma serine:glycine ratio might indicate decreased neurological aging [88,89] and decreased renal aging [90] in DFM hens.

### 3.5. BHMT Hyperactivation: A Means to Accelerate Hcy Remethylation (with the Additional Effect of Decreasing Liver Steatosis, Liver Mass, and Body Mass)

Our lab previously reported reduced liver steatosis, liver mass, and body mass in flaxseed-fed hens [91]. Those hens also had 80% reduced liver aspartate aminotransferase (AST) levels, indicating improved liver function. In our current study, our flaxseed-fed hens had reduced body mass concomitant with reduced plasma AST [92]. Researchers from a separate lab observed 50% reduced hepatic crude fat content when hens were fed 18% coextruded full-fat flaxseed [93], corroborating our finding of reduced liver fat content.

BHMT hyperactivation would not only accelerate Hcy remethylation, but it would also accelerate choline oxidation and PC catabolism, especially in the hepatocyte. Supporting this, we detected 39% lower choline levels and 14% reduced body mass, in WFX hens. BHMT hyperactivation would be expected to deteriorate the choline content, because choline is the only molecule that can replenish betaine. Meanwhile, betaine would be less likely to show decrease, because betaine is being replenished by choline (and hepatocytes specialize in storing betaine). Therefore, the “true catabolic hit” would be seen at the level of choline.

How could BHMT hyperactivation reduce liver steatosis, liver mass, and body mass? As choline becomes insufficient within hepatocytes, the cell must liberate additional soluble choline from PC (via phospholipases), or else the cell will suffer choline depletion [94]. The accelerated phospholipase activity will increase the production of non-esterified free fatty acids (FFAs). A portion of these FFAs will be oxidized via mitochondrial fatty acid oxidation (FAO). The tricarboxylic acid (TCA) cycle will then be recruited to oxidize the growing pool of FAO-derived acetyl CoA. Altogether, FAO and TCA cycling generate a large volume of “dissipative structure [95]”, specifically in the form of metabolic H_2_O, HCO_3_, CO_2_, and H^+^. When these structures exit the liver and/or body, the lipid mass of the liver and body will decline (assuming non-replacement).

### 3.6. Increased Input to the Folate Cycle: Also Accelerating Hcy Remethylation (Focus on DMG!)

We also suspect increased one-carbon input into the folate cycle, specifically via dimethylglycine dehydrogenase (DMGDH) and serine hydroxymethyltransferase (SHMT). DMGDH is the only enzyme in physiology known to catabolize DMG [42], increasing the likelihood that elevated DMGDH activity explains the lower DMG level observed in DFM hens and WFX hens. Increased DMGDH activity would also increase 5,10-CH_2_THF synthesis and thereby stimulate increased remethylation output via MS-B12. Reduced feed intake is unlikely to account for decreased DMG levels, particularly because DMG is derived from choline (and flaxseed is a rich source of choline).

DFM hens displayed slightly lower serine levels, on average, and a slightly lower serine:glycine ratio, particularly versus CRN hens. This might be interpreted as increased SHMT activity in DFM hens. Isotope-labeled serine flux could be employed to determine actual SHMT activity; however, our results might suggest elevated 5,10-CH_2_THF synthesis via SHMT in DFM hens. Furthermore, serine is a complex amino acid due to its ability to be highly metabolized in the kidneys as well as the liver.

Flaxseed is also the richest known source of the phytoestrogen secoisolariciresinol diglucoside (SDG), and SDG is the precursor for enterodiol (ED) and enterolactone (EL). We already know that ED and EL are highly elevated in DFM hens and WFX hens, according to a previous study from our lab [91]. ED and EL are phytoestrogens that are produced after the enteric fermentation of SDG [96]. In mice, ED and EL have been observed to exert mild estrogenic effects [97]. Future studies will evaluate the effects of ED and EL on BHMT activity and MS-B12 activity, in the laying hen, and this would help to further uncover the effects of flaxseed on one-carbon metabolism.

### 3.7. Flaxseed Could Decelerate Ovarian Tumor Metastasis by Reducing Omental Adiposity

The majority of clinically detected ovarian tumors are late-stage serous ovarian adenocarcinomas that are highly metastatic [98,99]. Fewer than 30% of women with these late-stage ovarian tumors will survive beyond five years of their initial diagnosis, primarily due to the fact of metastatic spread and chemoresistance [100]. Ovarian tumors almost always metastasize to a white adipose tissue known as the omentum [101]. Obesity is a risk factor for ovarian cancer metastasis to the omentum, because increased fat deposition in the omentum enhances adipokine signaling (which drives tumor metastasis) [102].

By reducing omental fat deposition, flaxseed would decrease adipokine signaling and decelerate ovarian tumor metastasis. In our current work, we observed 9–14% reduced body mass in flaxseed-fed hens. Our lab previously observed similar mass reduction results [91]. Flaxseed’s mass reducing effects are just as effective in humans. A meta-analysis of 45 randomized controlled trials concluded that whole flaxseed (>30 g/d) significantly lowers body weight, body mass index (BMI), and waist circumference with the biggest benefits in obese human subjects [103]. We predict that consistently consumed whole flaxseed should reduce omental adiposity and thereby decrease the inflammatory crosstalk between the omentum and the ovarian tumor. In this manner, flaxseed could slow tumor progression and/or help women to remain in remission from ovarian cancer.

A gynecologic oncology professor once shared that, “it is as though flaxseed causes the ovarian tumor to lose its appetite for fat” (David Huntsman, personal communication). The hypothesis that flaxseed protects women from ovarian tumor metastasis by reducing omental fat deposition, supports Huntsman’s notion.

### 3.8. Flaxseed Protects Individuals from Cachexia during Cancer, by Improving Liver Function

Cancer patients commonly suffer from a wasting syndrome known as cachexia, where the body excessively catabolizes protein mass (sarcopenia) and lipid mass [104,105]. Approximately 22–40% of cancer patients die from cachexia-associated complications [106,107]. Cachexia, being a systemic imbalance, challenges multiple organs of the body including the liver [108]. Cachexia-induced liver dysfunction is similar to the effects of cirrhosis [109], where hepatocytes cannot viably perform duties such as VLDL secretion, gluconeogenesis, xenobiotic metabolism, and bile secretion [28,108,110,111].

Previous results from our lab indicate that flaxseed boosts liver function in hens [91,92]. We predict that BHMT hyperactivation and PEMT hyperactivation empower flaxseed with the ability to protect the animal (or individual) from cancer-associated liver dysfunction, and this directly protects the animal from the wasting effects of cachexia. From a pre-clinical perspective, this might be why flaxseed is an excellent choice to protect cancer patients. By alleviating the effects of cachexia, flaxseed could improve patient survival and improve patient quality of life. Optimal liver function should also promote systemic metabolic homeostasis, given the plethora of metabolic tasks that the liver must perform. We observed that the body masses of normal hens and cancerous hens were similar within the DFM diet and WFX diet, suggesting the possibility of metabolic homeostasis (independent of disease state) in the animals that we already know have improved liver function (i.e., [91]).

### 3.9. Flaxseed Likely Regulates the Methyltransferome by Augmenting One-Carbon Metabolism

WFX10 hens had a high ratio of downregulated-to-upregulated array features in both normal and cancerous ovaries. Chromosomal architecture within tumors is chaotic due to the increased expression of DNA methyltransferases (e.g., DNMT3A) and histone methyltransferases (e.g., PRMT5) [81,112]. Our study shows that flaxseed likely overcomes (to an extent) the chaotic environment of ovarian tumors by exerting a downregulatory effect on the transcriptome of the ovarian tumor.

In normal ovaries, we observed numerous SAM-dependent array features that were downregulated by the WFX10 diet. Among these features were methyltransferases that act on histone-lysine residues, tRNAs, rRNA, mRNA, and DNA, suggesting that the WFX10 diet augments the methyltransferome of the ovary. Future studies will investigate how flaxseed augments histone-lysine methylation (e.g., H3K4-me) in laying hens, especially because KMT2C was among the most heavily downregulated features in the microarray. Other researchers have argued that histones serve as “SAM sinks” when SAM levels are high [30], meaning that histones become hypermethylated in the presence of excess SAM. This could be the case for flaxseed-fed hens; however, these animals have excess MTA to protect against excessive hypermethylation. Future studies will evaluate the interplay between SAM and MTA to determine how histone methylation is regulated in flaxseed-fed hens.

### 3.10. Comprehensive Model of One-Carbon Metabolism in Flaxseed-Fed Hens

**Figure 10:**
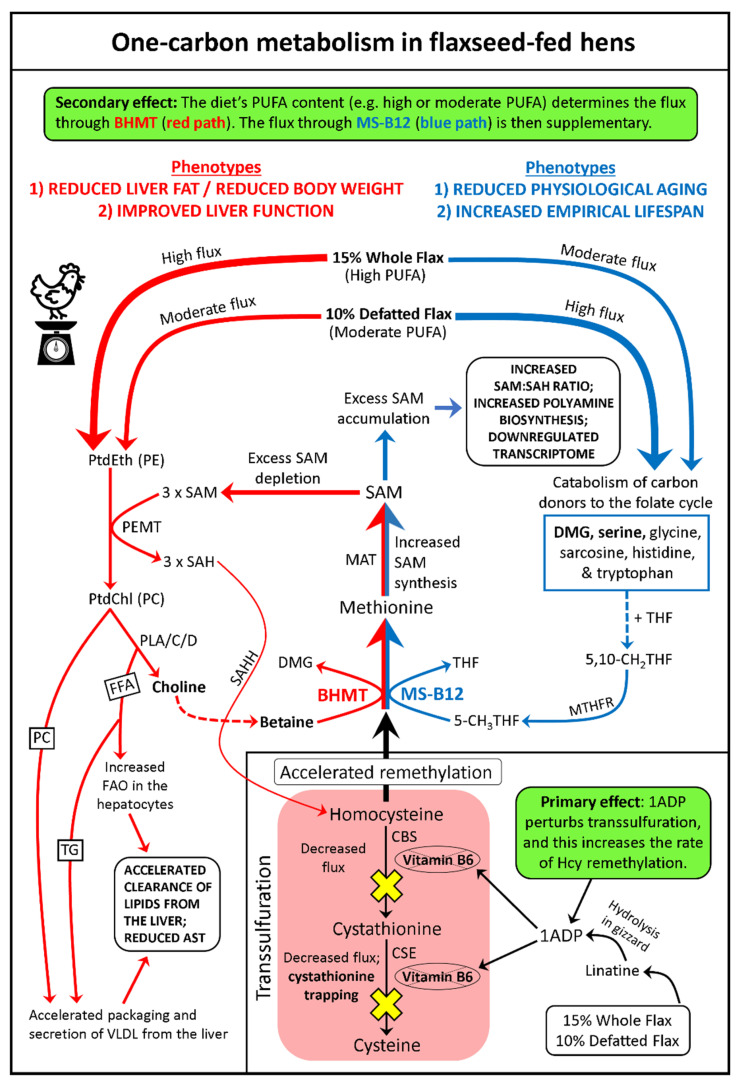
Comprehensive model of one-carbon metabolism in flaxseed-fed hens. The **primary metabolic effect** (transsulfuration perturbation) is initiated when lADP exerts an anti-vitamin B6 effect that reduces CBS and CSE activity in the liver. This reduces transsulfuration flux and drives accelerated remethylation of Hcy into the Met cycle. This culminates in the increased synthesis of SAM, so that WFX hens and DFM hens have access to excess SAM. The **secondary metabolic effect** (flux through BHMT) is determined by the PUFA content of the diet, such that WFX hens exhibit a high flux through BHMT, and DFM hens exhibit a moderate flux through BHMT. The high PUFA content of the WFX diet allows WFX hens to strongly hyperactivate PEMT, while the moderate PUFA content of the DFM diet allows DFM hens to only moderately hyperactivate PEMT. In WFX hens, all excess SAM is catabolized by PEMT, because these animals strongly hyperactivate PEMT. In contrast, DFM hens only moderately hyperactivate PEMT and, therefore, not all excess SAM will be catabolized. This means that some of the excess SAM will accumulate in DFM hens and culminate in an increased plasma SAM:SAH ratio (i.e., increased methylation index). Simultaneously, this will yield increased production of MTA, due to enhanced flow of SAM toward polyamine biosynthesis. The elevated SAM:SAH ratio, in concert with elevated MTA, then acts to epigenetically downregulate transcriptomic processes in flaxseed-fed hens. By downregulating the transcriptome of the animal, this reduces physiological aging and culminates in increased empirical lifespan. One of the main themes of this model is that many things are accelerated, in order to compensate for reduced flux through transsulfuration. For example, accelerated flux through BHMT causes increased betaine catabolism, and therefore, causes increased choline oxidation. In turn, the hepatocyte must accelerate the phospholipase-mediated catabolism of PC, so that the hepatocyte can maintain access to sufficient levels of choline. This phospholipase activity generates a large quantity of FFAs that must be oxidized via mitochondria FAO, which contributes to the accelerated clearance of lipids from the liver. The oxidation of these FFAs also contributes to reduced adiposity and reduced body weight. Some of these FFAs will be incorporated into TG and packaged into VLDL, and this will assist with further clearance of lipids from the liver. Another major contributor to reduced hepatic steatosis is PEMT hyperactivation. By increasing PEMT activity in hens, flaxseed accelerates the synthesis of PC, and PC is a rate limiting molecule for the packaging and secretion of VLDL from the liver. Accelerated secretion of VLDL from the liver accelerates the clearance of lipids from hepatocytes and culminates in reduced hepatic steatosis. Without question, this would improve liver function (as evidenced by reduced hepatic AST in our flaxseed-fed hens). The flux through MS-B12 was supplementary to the flux through BHMT, such that WFX hens exhibited a moderate flux through MS-B12 while DFM hens exhibited a high flux through MS-B12. DFM hens and WFX hens likely displayed increased catabolism of DMG via DMGDH, indicating that both diet groups had elevated synthesis of 5,10-CH_2_THF. DFM hens also likely displayed elevated catabolism of serine via SHMT, as evidenced by a slightly lower serine:glycine ratio, further indicating elevated 5,10-CH_2_THF synthesis. Notably, this model predicts that the excess single-carbon units from serine (within the folate cycle, via SHMT) act as a unique carbon source permitting the accumulation of SAM. This suggests that serine is a lifespan extending amino acid in hens, and that SHMT is a lifespan extending enzyme. 1ADP = 1-amino d-proline; 5,10-CH_2_THF = 5,10 methylene tetrahydrofolate; AST = aspartate amino transferase; BHMT = betaine homocysteine methyltransferase; CBS = cystathionine beta synthase; CSE = cystathionase; dcSAM = decarboxylated SAM; DMG = dimethylglycine; DMGDH = dimethylglycine dehydrogenase; FAO = fatty acid oxidation; FFA = free fatty acid; Hcy = homocysteine; MAT = methionine adenosyltransferase; MS-B12 = methionine synthase-B12; MTA = methylthioadenosine; MTHFR = methylene tetrahydrofolate reductase; PC = phosphatidylcholine; PUFA = polyunsaturated fatty acid; PEMT = phosphatidylethanolamine methyltransferase; SAH = S-adenosylhomocysteine; SAM = S-adenosylmethionine; SHMT = serine hydroxymethyltransferase; TG = triglyceride; VLDL = very low-density lipoprotein.

We created a comprehensive model of flaxseed’s effects on one-carbon metabolism, in association with endpoint biological outcomes in the laying hen (Figure 10). This model provides a network illustration of our empirical observations and hypothetical insights. Within the model, we encourage the reader to observe two major processes. The first major process is flaxseed’s “primary effect” that is facilitated through 1ADP’s anti-B6 effect on transsulfuration flux. The other major process is flaxseed’s “secondary effect” that determines the hen’s increased carbon flux through either BHMT (red path) or MS-B12 (blue path). This “branching point” is determined by the PUFA content of the diet. Ultimately, we observed that BHMT was mostly associated with weight loss and improved liver function, whereas the flux through MS-B12 was associated with decreased physiological aging and increased empirical lifespan.

## 4. Conclusions

We have demonstrated that 1ADP is a driver behind flaxseed’s main effects, at least in birds. This study indicated that flaxseed, via the anti-B6 effect of 1ADP, redirects carbon flux through one-carbon metabolism in a manner that increases lifespan and reduces ovarian cancer severity in laying hens. Our model suggests that 1ADP reduces transsulfuration flux in an anti-B6 manner, culminating in accelerated remethylation of Hcy (via BHMT and MS-B12). We have evidence that flaxseed increases the consumption of one-carbon donor molecules (i.e., choline, betaine, DMG, and serine) that fuel BHMT and MS-B12. In support of BHMT hyperactivation and MS-B12 hyperactivation, we observed elevated SAM, an elevated SAM:SAH ratio, and elevated MTA, in flaxseed-fed hens. Altogether, this supports the hypothesis that the anti-B6 effects of flaxseed accelerate Hcy remethylation, SAM synthesis, and polyamine biosynthesis in hens.

The associated biological outcomes in these animals are increased lifespan, reduced physiological aging, improved liver function, and decreased body mass. Lifespan was enhanced optimally by the DFM diet, while liver function and body leanness were enhanced optimally by the WFX diet.

The augmented one-carbon metabolome of a flaxseed-fed hen likely has the ability to influence epigenetic processes (i.e., histone-lysine methylation) in extrahepatic tissues such as the ovary. Our reanalysis of microarray features in the ovary suggests that flaxseed exerts a general downregulation of the transcriptome in both normal and cancerous ovaries, with a pronounced effect on SAM-dependent methylatransferases. It seems plausible that this global effect is the result of an elevated SAM:SAH ratio and an elevated level of MTA. Future work will more rigorously examine flaxseed’s effect on the laying hen methylome (e.g., histone methylation, DNA methylation, and RNA methylation) and evaluate these effects in the context of aging, lifespan, and cancer.

Our model predicts that BHMT hyperactivation plays a key role in determining weight loss and liver health in flaxseed-fed hens. Simultaneously, MS-B12 hyperactivation appears to be a major contributor to reduced aging and increased lifespan. We also predict that flaxseed induces PEMT hyperactivation, with greater activation stemming from flaxseed diets that are highly enriched with PUFAs (i.e., 15% whole flaxseed).

By improving liver function, flaxseed can help to protect cancer patients from the body wasting effects of cachexia. A myriad of metabolic processes in the body are dependent upon proper liver function (e.g., VLDL secretion, gluconeogenesis, and bile secretion). As such, flaxseed is an excellent choice to improve liver function in cancer patients. In turn, these individuals will suffer less from cancer-associated cachexia. Flaxseed is also an excellent choice for reducing fat deposition within the omentum. By reducing omental adiposity, flaxseed can reduce the rate of ovarian tumor metastasis. This would provide a specific means by which flaxseed protects women from late-stage ovarian cancer. Simply put, flaxseed is a nutritional best friend for hens and for ovarian cancer patients.

## 5. Materials and Methods

### 5.1. Animal Studies and Diet Descriptions

Single-comb White Leghorn laying hens (sp. *Gallus gallus*) with 2.5 years of age were assigned to one of six isocaloric diets for 325 days. The ingredients of these diets as well as their calculated energy contents can be seen in Table 3 and Table 4, respectively. Sample sizes of hens assigned to each diet were as follows: CTL (*n* = 182), DFM (*n* = 161), WFX (*n* = 161), FXO (*n* = 161), CRN (*n* = 175), and FSH (*n* = 165)**.** We also mention a previously published diet study where 2.5-year-old White Leghorn hens were fed either a 10% whole flaxseed diet (WFX10, *n* = 193 hens) or a control diet (CTL, *n* = 193 hens) for one year [11]. Note that all references of “WFX” were to the 15% whole flaxseed diet. Hens were housed in the animal care facility at the University of Illinois in Urbana-Champaign, in groups of 5 hens with a 17 h/7 h light/dark cycle. Daily mortality was monitored by the animal care facility; however, the causes of death were not investigated. Feed and water were not restricted at any point during studies. SIUC animal protocol approval assurance number D16-0004, approved by IACUC on 23 April 2020.

### 5.2. Plasma Collection and Animal Necropsy

Blood plasma was isolated, as follows. Hens were bled via wing vein puncture on the 220th day of the study, and blood was transferred to citrate-treated tubes. Tubes were then centrifuged at 2000 rpm for 10 min at 4 °C, and the supernatant (plasma) was transferred to fresh tubes and stored at −80 °C. All hens that survived to the completion of the study had their body weight recorded prior to CO_2_ asphyxiation and cervical dislocation. Body weight was recorded for the following number of “normal” hens: CTL = 86, DFM = 85, WFX = 72, FXO = 84, CRN = 62, and FSH = 70. Similarly, body weight was recorded for the following number of “cancerous” hens: CTL = 31, DFM = 23, WFX = 22, FXO = 22, CRN = 29, and FSH = 29. In total, we observed 459 normal hens and 151 cancerous hens. The peritoneal cavity of each hen was investigated to obtain visual descriptions of the cancer involvement, and biological samples of the peritoneal cancers (i.e., ovarian cancer, gastrointestinal cancer, liver cancer, and oviductal cancer) were obtained and flash frozen in liquid nitrogen. All samples are kept at −80 degrees Celsius at Southern Illinois University, in Carbondale, IL, USA.

### 5.3. LC-MS/MS Analysis of Plasma Metabolites

Plasma samples were packaged in dry ice and transferred to the Lipid and Metabolite Mass Spectrometry Facility of the University of Texas Southwestern Medical Center, for analysis via liquid chromatography tandem mass spectrometry (LC-MS/MS). Sample sizes for this analysis were CTL (*n* = 6), DFM (*n* = 5), WFX (*n* = 6), FXO (*n* = 6), CRN (*n* = 6), and FSH (*n* = 4). The LC-MS/MS procedure was described in detail [113]. In brief, a targeted metabolite profiling approach was conducted by separating metabolites on a Phenomenex Synergi Polar-RP HPLC column with a Nexera Ultra High-Performance Liquid Chromatography (UHPLC) system (Shimadzu, Kyoto, Japan). An AB QTRAP 5500 mass spectrometer (Applied Biosystems SCIEX, Foster City, CA, USA) was used with electrospray ionization (ESI) source set to multiple reaction monitoring (MRM) mode. MRM data were acquired using Analyst 1.6.1 software (Applied Biosystems SCIEX, Foster City, CA, USA). Chromatograms and peak areas were measured with MultiQuant 2.1 software (Applied Biosystems SCIEX, Foster City, CA, USA). Peak areas were normalized for each detected metabolite against the total ion count of that sample to correct for variation introduced by sample handling. Peak areas were converted to VIP scores. VIP scores, heat map, and partial least squares-discriminant analysis (PLS-DA), were calculated using the software SIMCA-P (Version 13.0.1, Umetrics, Umeá, Sweden). VIP scores are arbitrary units that are not representative of translatable concentrations.

### 5.4. RNA Isolation and cDNA Synthesis

In brief, RNA was collected from hen liver homogenates using the Trizol^TM^ method with chloroform phase separation [114]. RNA quality was determined via electrophoresis on a 1% agarose formaldehyde gel, using ethidium bromide as a fluorescent stain. For cDNA synthesis, 2 µg of RNA was used for each sample and then incubated with DNAse for 30 min at 37 °C and with DNAse stop solution for 10 min at 65 °C. Reverse transcriptase master mix was then prepared, using (per sample) 3.2 µL of nuclease-free water, 2 µL of 10X reverse transcriptase buffer, 2 µL of 10X random primers, 1 µL of Multiscribe^TM^ reverse transcriptase (Applied Biosystems, 4311235), and 0.8 µL of 100 mM dNTP mix. Buffer, primers, and dNTP mix were included as a kit (A&B Biosystems, 4368814). The 20 µL cDNA samples were then incubated at 25 °C (10 min), 37 °C (90 min), 37 °C (30 min), 85 °C (5 min), and cooled to 4 °C.

### 5.5. qPCR Analysis

This qPCR protocol uses 10 µL reactions in a Bio-Rad CFX384^TM^ thermocycler (Bio-rad, Hercules, CA, USA). Each reaction received 0.1 µL of 100 µM forward/reverse oligonucleotide primer, 5 µL of SsoFast^TM^ EvaGreen^®^ Supermix (Bio-rad, 1725203), 3.8 µL of nuclease-free water, and 1 µL of cDNA template. The qPCR protocol included activation of EvaGreen at 95 °C for 3 min; denaturation of cDNA at 95 °C for 15 s; annealing and extending at 60.4 °C. This protocol was carried out for 40 rounds. All Ct values and melt peaks were analyzed using the CFX Manager^TM^ software (Bio-rad, Hercules, CA, USA). qPCR data were analyzed using the 2(-Delta Delta C(T)) method as described in [115]. Glyceraldehyde 3-phosphate dehydrogenase (GAPDH) was used as a reference gene. The oligonucleotide primers that were used for qPCR are shown in Table 5.

### 5.6. Reanalysis of Microarray Features Expressed in Hen Ovaries

Microarray analysis was previously conducted [9] on ovarian tissues (normal and cancerous) of hens that were fed either WFX10 or CTL in order to measure the expression of 44,000 features using an Agilent custom chicken long oligo microarray. The purpose of the microarray was to compare the effects of CTL or WFX10 on feature expression in normal and cancerous hen ovaries. Basically, the research design required two-tailed *t*-test comparisons of feature expression (i.e., WFX10 normal versus CTL normal or WFX10 cancer versus CTL cancer). Sample size was *n* = 6 per diet/disease group. In our present paper, we reanalyzed feature expression from that data set and reported the percentage of features that were downregulated or upregulated by WFX10 at *p* < 0.1, *p* < 0.05, and *p* < 0.01 levels of significance from *t*-tests.

### 5.7. Statistical Analysis

Statistical analysis was performed with R statistical software (version 3.6.3; r-project.org). Significant differences between diet groups were determined via one-way ANOVA and Duncan’s Multiple Range post-test (Duncan’s significant at *p* < 0.05). We included a supplementary table (see Supplementary Materials, Table S3) with the F-test *p*-values of all one-way ANOVAs that were conducted in the main body of this research. When Bartlett’s statistic was significant at *p* < 0.05, the data were log_10_ transformed prior to conducting ANOVA. During the analysis of plasma metabolites, we removed significant outliers using a z-score method of detecting outliers at *p* < 0.05. Dendrogram clustering analysis was conducted via R package “dendextend”, using a default k-means algorithm (k = 6). Kaplan-Meier survival analysis and Cox proportional hazard (Coxph) analysis were conducted via the R packages “survival” and “survminer” (*p* < 0.05). A two-tailed *t*-test (assuming unequal variance) was also conducted during body weight analysis (*p* < 0.05).

## Figures and Tables

**Table 1:** Microarray feature expression in laying hen ovaries (WFX10 versus CTL).

Ovarian Tissue Type	*p*-Value Cutoff from *t*-Test (Whole Flax Versus Control Diet)	Total Number of Features Affected at *p*-Value Cutoff			Ratio of Downregulated to Upregulated	Average Fold Change Per Downregulated or Upregulated Feature	
		Total (*n*)	* Down by WFX10 (*n*)	^#^ Up by WFX10 (*n*)	Ratio (Down/Up)	Fold Down	Fold Up
Normal ovary	*p* < 0.10	5042	3479	1563	2.23	−1.74	+1.53
	*p* < 0.05	2198	1671	527	3.17	−1.83	+1.64
	*p* < 0.01	156	127	29	4.38	−2.09	+1.91
Cancerous ovary	*p* < 0.10	2965	1721	1235	1.39	−1.60	+1.60
	*p* < 0.05	1199	767	432	1.78	−1.75	+1.66
	*p* < 0.01	157	120	37	3.24	−2.01	+1.90

* Down = downregulated; ^#^ Up = upregulated.

**Table 2:** Microarray features related to SAM-dependent metabolism, *p* < 0.01 (WFX10 versus CTL).

Ovarian Tissue Type	Gene Name	Description of Gene	Fold Change	*p*-Value
Normal ovary	KMT2C	Histone-lysine methyltransferase (H3K4 methyltransferase)	−2.10	0.005
	TRMT1L (C1orf25)	tRNA methyltransferase	−2.10	0.007
	SAM-MT	Conserved sequence domain for an unnamed SAM-dependent methyltransferase	−2.04	0.008
	NOL1, NOP2	Ribosomal RNA methyltransferase	−1.97	0.009
	CBWD1 (COBW)	Cobalamin (B12) biosynthesis	−1.88	0.009
	RSBN1 (ROSBIN)	Histone-lysine demethylase (H4K20 demethylase)	−1.67	0.005
Cancerous ovary	SLC25A26	Mitochondrial SAM carrier protein (SAMC)	+2.07	0.003

**Table 3:** Diets and associated ingredients (g/100 g of diet).

Ingredient (g/100 g)	Control (CTL)	10% Defatted Flaxseed Meal (DFM)	15% Whole Flaxseed (WFX)	5% Flax Oil (FXO)	5% Corn Oil (CRN)	5% Menhaden Fish Oil (FSH)
Corn	67.40	54.90	47.58	52.00	52.00	52.00
Soybean meal	18.30	18.30	18.30	18.30	18.30	18.30
Flaxseed (whole)			15.00			
Corn Gluten Meal	3.00			5.00	5.00	5.00
Corn Oil					5.00	
Flax Oil				5.00		
Fish Oil						5.00
Defatted Flax Meal		10.00				
Qual Fat		3.80	2.50			
Solka Floc	0.30	2.00	5.62	8.70	8.70	8.70
Each diet received the following in g/100 g of diet: limestone (8.75), dical (1.5), salt (0.3), vitamin mix ^1^ (0.2), mineral mix ^2^ (0.15), and dl-methionine (0.1).						

^1^ Vitamin premix (per kg of diet): retinyl acetate, 4400 IU; cholecalciferol, 25 mg; dl-a-tocopheryl. acetate, 11 IU; vitamin B12, 0.01 mg; riboflavin, 4.41 mg; d-Capantothenate, 10 mg; niacin, 22 mg; and menadione sodium bisulfite, 2.33 mg. ^2^ Mineral premix (mg/kg of diet): Mn, 75 from MnO; Fe, 75 from FeSO_4_·7H_2_O; Zn, 75 from ZnO; Cu, 5 from CuSO_4_·5H_2_O; I, 0.75 from ethylene diamine dihydroiodide; and Se, 0.1 from Na_2_SeO_3_.

**Table 4:** Calculations of nutrient percentages and total energy of diets.

Calculated Analysis	Control (CTL)	10% Defatted Flaxseed Meal (DFM)	15% Whole Flaxseed (WFX)	5% Flax Oil (FXO)	5% Corn Oil (CRN)	5% Menhaden Fish Oil (FSH)
TME ^1^, kcal/kg	2816	2816	2815	2815	2815	2815
CP ^2^, % TME	16.56	17.04	16.50	16.49	16.49	16.49
Calcium, % TME	3.73	3.77	3.75	3.73	3.73	3.73
aPhosphorus ^3^, % TME	0.38	0.40	0.38	0.37	0.37	0.37
Met + Cys, % TME	0.67	0.72	0.64	0.67	0.67	0.67

^1^ TME = total metabolizable energy; ^2^ CP = crude protein; ^3^ aPhosphorous = available phosphorous.

**Table 5:** Oligonucleotide primers used in this study (sp. *Gallus gallus*).

Gene	Forward Primer (5′ to 3′)	Reverse Primer (5′ to 3′)
BHMT	TGGGTCAGAGCAAGAGCAAGAAA	TGGTCACTCCCCAGCCATCT
CBS	GTTCCAGTGTCTAAGGCCAAGCC	CTTACAGTCCTCAGTGTCTGTCCCA
CSE	CACTTCGGCACGCAGGCCAT	CGCCTGCTGCTTGAACGTGGT
GAPDH	ACAGCAACCGTGTTGTGGAC	CAACAAAGGGTCCTGCTTCC
MS	CAGAGCCGCAGAAGAAAGCAAG	AGAGGTGTGCCTCGGAAGTGA
MTHFR	GGCAGCAGCAGTGGGAGTG	GCCTCCGCCGCATCTTCTC

## Data Availability

Microarray data are MIAME compliant and available in Gene Expression Omnibus (GEO, http://www.ncbi.nlm.nih.gov/geo/, accessed on 15 June 2019) through the accession number GSE40376. All other data presented in this article are available on request from the corresponding author.

## Supplementary Materials

The following are available online, Figure S1. Expanded model illustrating one-carbon metabolism (minus transsulfuration); Figure S2. Partial least squares discriminate analysis (PLSDA); Figure S3. Plasma estimate of renal clearance; Figure S4. Plasma estimate of creatine metabolism and GAMT activity; Figure S5. BHMT, MS, and MTHFR gene transcript levels in liver homogenates of hens; Figure S6. Dendrogram showing K-means clustering of one-carbon donating molecules; Table S1. Plasma levels of molecules that negatively regulate BHMT in hens, by diet group; Table S2. Cox proportional hazard analysis of hen survival, using the corn oil diet as reference; Table S3. F-test results (*p*-values) from one-way ANOVAs that were conducted in Figure 3, Figure 4, Figure 5, Figure 6 and Figure 7.

## Sample Availability

Samples of the diets are available from the corresponding author.

## Abbreviations

**Abbreviation**	**Full term**
4PA	4-Pyridoxic Acid
B6	Vitamin B6
5,10-CH_2_THF	5,10-Methylene Tetrahydrofolate
5-CH_3_THF	5-Methyl Tetrahydrofolate
BADH	Betaine aldehyde dehydrogenase
BHMT	Betaine homocysteine methyltransferase
CBWD1	Cobalamin Synthetase W Domain-Containing Protein 1
CHDH	Choline dehydrogenase
CRN	5% Corn Oil
CTL	Control Diet
DNMT3A	DNA methyltransferase 3A
*dc*SAM	Decarboxylated SAM
DFM	10% Defatted Flaxseed Meal
DHA	Docosahexaenoic Acid
DMG	Dimethylglycine
DMGDH	Dimethylglycine dehydrogenase
ED	Enterodiol
EL	Enterolactone
EPA	Eicosahexaenoic Acid
FSH	5% Fish Oil
FXO	5% Flaxseed Oil
GAPDH	Glyceraldehyde 3-phosphate dehydrogenase
GCS	Glycine Cleavage System
GPC	Glycerophosphorylcholine
Hcy	Homocysteine
HHcy	Hyperhomocysteinemia
KMT2C	Lysine methyltransferase 2C
MAT	Methionine adenosyltransferase
Met	Methionine
MS-B12	Methionine synthase (complexed with B12)
MTA	Methylthioadenosine
MTHFR	Methylene tetrahydrofolate reductase
PC	Phosphatidylcholine
PE	Phosphatidylethanolamine
PEMT	Phosphatidylethanolamine methyltransferase
PLA	Phospholipase A
PLC	Phospholipase C
PLD	Phospholipase D
PRMT5	Protein arginine methyltransferase 5
PLP	Pyridoxal 5′ Phosphate
PUFA	Polyunsaturated Fatty Acid
SAH	S-adenosylhomocysteine
SAHH	S-adenosylhomocysteine hydrolase
SAM	S-adenosylmethionine
SDG	Secoisolariciresinol diglucoside
SDH	Sarcosine Dehydrogenase
SEM	Standard error of the mean
SHMT1	Serine hydroxymethyltransferase 1 (cytosolic SHMT)
SHMT2	Serine hydroxymethyltransferase 2 (mitochondrial SHMT)
TG	Triglyceride
THF	Tetrahydrofolate
TME	Total Metabolizable Energy
VIP	Variable Importance of Projection
VLDL	Very Low-Density Lipoprotein
WFX	15% Whole Flaxseed
WFX10	10% Whole Flaxseed
