# Supplementary material for: Flaxseed Increases Animal Lifespan and Reduces Ovarian Cancer Severity by Toxically Augmenting One-Carbon Metabolism
Source: Molecules. 2021 Sep 18;26(18):5674. doi: 10.3390/molecules26185674 (PMC8471351; doi:10.3390/molecules26185674)
Supplement: Supplementary file 1 [file molecules-26-05674-s001.zip › molecules-1315327-supplementary.pdf]

Supplementary Information

Flaxseed Increases Animal Lifespan and Reduces Ovarian Cancer Severity by Toxically Augmenting One-Carbon Metabolism

William C. Weston[1], Karen H. Hales[2], Dale B. Hales[1,2,*]

Table of Contents

Figures

Figure S1. Expanded model illustrating one-carbon metabolism (minus transsulfuration).

Figure S2. Partial least squares discriminate analysis (PLSDA).

Figure S3. Plasma estimate of renal clearance.

Figure S4. Plasma estimate of creatine metabolism and GAMT activity.

Figure S5. BHMT, MS, and MTHFR gene transcript levels in livers.

Figure S6. Dendrogram showing K-means clustering of one-carbon donating molecules.

Tables

Table S1. Plasma levels of molecules that negatively regulate BHMT, in hens by diet group.

Table S2. Cox proportional hazard analysis of hen survival, using the corn oil diet as reference.

Table S3. F-test results (p-values) from One-way ANOVAs that were conducted in Figures 3, 4, 5, 6, and 7.

Supplementary Information

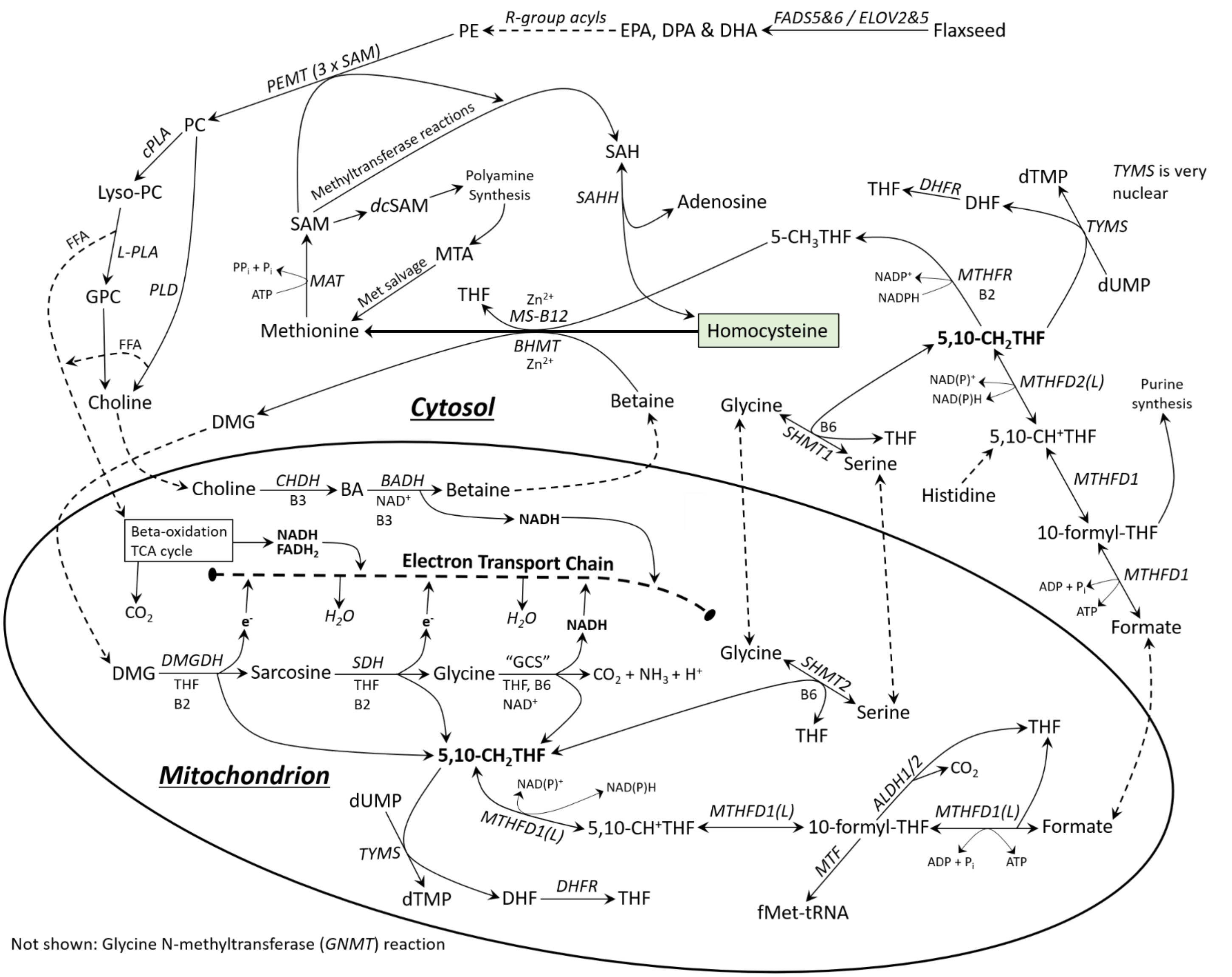

Figure S1. Expanded model illustrating one-carbon metabolism (minus transsulfuration). The primary focus of this model is to illustrate the folate cycle, methionine cycle, and phospholipid metabolism, according to their integration into one-carbon metabolism. Animals create Met by methylating Hcy via the activity of BHMT and MS-B12 [1,2]. BHMT is also a zinc-dependent metalloenzyme, but it utilizes betaine to methylate Hcy [3]. Endogenously, betaine is derived from the irreversible oxidation of choline, and the majority of choline is derived from the catabolism of phosphatidylcholine (PC). Flaxseed, as a rich source of polyunsaturated fatty acids (PUFAs), contributes to the synthesis of phosphatidylethanolamine (PE). PEMT is the only enzyme known in biology to provide *de novo* choline synthesis, by way of converting phosphatidylethanolamine (PE) to PC. PEMT synthesizes PC from PE by using three S-adenosylmethionine (SAM) molecules to tri-methylate PE [4]. The rate of PEMT activity is dependent on the concentration of PEMT's two primary substrates, PE and SAM [5,6]. In the liver, BHMT facilitates 50% of Hcy methylation; the other 50% is facilitated by MS-B12 [7–10]. MS-B12 is a zinc-dependent matalloenzyme that utilizes 5-CH_3THF as a methyl donor to remethylate Hcy [11]. MS-B12 is expressed in all tissues throughout the body, whereas BHMT expression is mainly restricted to the liver and kidney [3,12]. Once Met is formed by BHMT or MS-B12, it can be converted to S-adenosylmethionine (SAM) by methionine adenosyl transferase (MAT) [13]. The liver is the primary organ of SAM synthesis given that 50% of all methionine metabolism and 85% of all methyltransferase reactions occur hepatically [13,14]. SAM is the master methyl donor, because it is required for a myriad of methyltransferase reactions, including PC synthesis [15], creatine synthesis [14], sarcosine synthesis [16], histone protein methylation [17], 5-methylcytosine DNA or RNA methylation [18,19], N6-methyladenosine RNA methylation [20], catechol structure methylation [21], and other targets. SAM becomes S-adenosylhomocysteine (SAH) when it donates its methyl group to a substrate molecule. **(legend continued on the next page)**

(legend continued) SAH, having structural similarity to SAM, is a potent allosteric inhibitor of most SAM-dependent methyltransferases [22]. The enzyme S-adenosylhomocysteine Hydrolase (SAHH) is responsible for hydrolyzing SAH into Hcy and adenosine, making SAHH critical for the sustainment of SAM-dependent methyltransferase reactions. This is why the SAM:SAH ratio, also known as the 'methylation index', is important for determining the animal's capacity to activate SAM-dependent methyltransferases [23,24]. A higher methylation index generally indicates increased capacity for methyltransferase activity. The folate cycle is crucial for supporting a myriad of physiological reactions, including reactions that sustain the methylation capacity of the animal. The folate cycle becomes biologically active when tetrahydrofolate (THF) receives a one-carbon group such as formyl group (-HCO), methylene group (CH_2) or methyl group (CH_3), to yield 10-formyl-THF, 5,10-CH_2THF or 5-CH_3THF, respectively [25]. 5,10-CH_2THF is the substrate for numerous enzymes such as Methylene Tetrahydrofolate Reductase (MTHFR), Serine Hydroxymethyltransferase (SHMT), Thymidylate Synthase (TYMS), and bidirectional Methylene Tetrahydrofolate Dehydrogenase (MTHFD2) [25]. MTHFR is a cytosolic FAD-dependent enzyme that uses NADPH to reduce 5,10-CH_2THF to form 5-CH_3THF. 5-CH_3THF is then used by MS-B12 to methylate Hcy to form Met [26], thereby supporting the methylation index of the animal [27]. SHMT includes a cytosolic (SHMT1) and a mitochondrial (SHMT2) isoform; both of which are B6-dependent enzymes that consume serine and THF in a reversible reaction that yields 5,10-CH_2THF and glycine [28,29]. Serine, via SHMT, is the cell's most important contributor to the synthesis of 5,10-CH_2THF. Several other substrates and enzymes contribute directly to the synthesis of 5,10-CH_2THF. Those substrate/enzyme pairings include: dimethylglycine (DMG) via Dimethylglycine Dehydrogenase (DMGDH), sarcosine via Sarcosine Dehydrogenase (SDH), and glycine via the 4-protein Glycine Cleavage System ("GCS") [30,31].

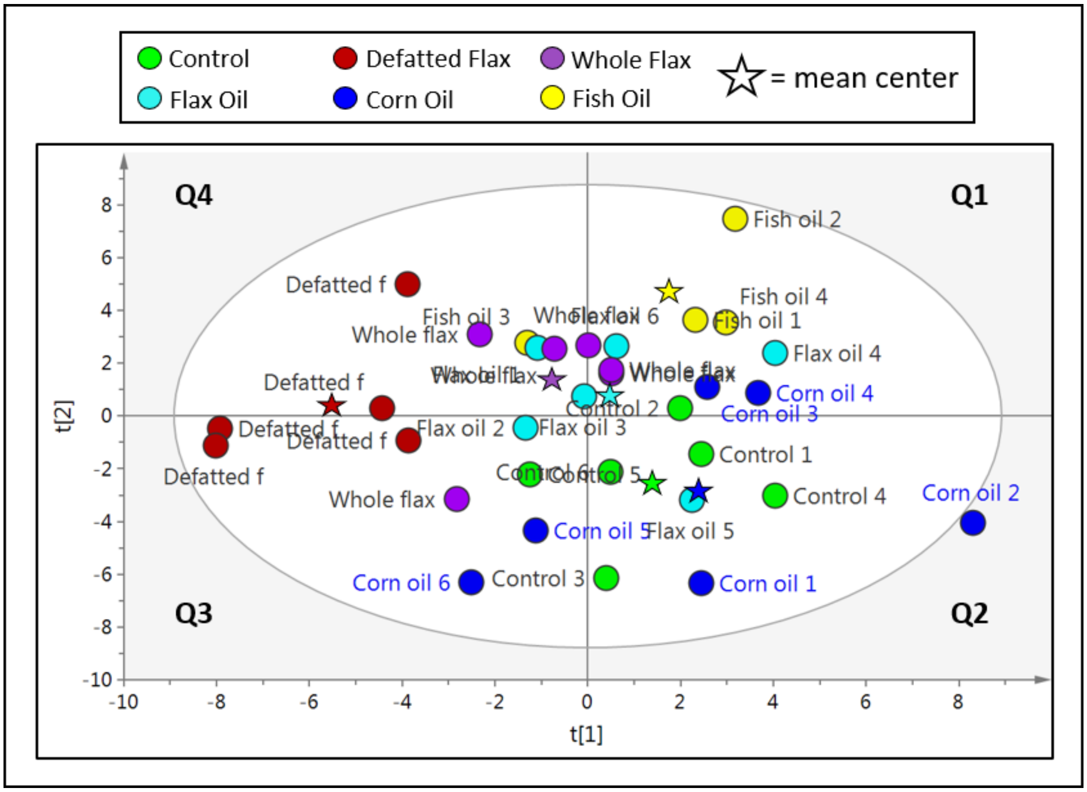

Figure S2. Partial least squares discriminate analysis (PLSDA). VIP scores from the metabolites were used to cluster plasma samples according to PLSDA analysis. WF hens displayed the highest degree of clustering (notably, two WF samples perfectly coincident in Q1), followed by DF, FSH, FXO and CTL. DF hens were the most unique in terms separation from all other diets. Dietary enrichment with flaxseed oil (ie WF and FXO) caused a high degree of two-dimensional overlap. CRN was the least clustered, displaying a pattern of dispersion across Q1, Q2 and Q3. The mean center (ie the average XY dimension) for WF was located in Q4 near the origin (x=0, y=0). Similarly, FXO's mean center was also near the origin. DF's mean center showed a strong left shift beyond all other diets and was placed slightly into Q4. The mean centers of WF, FXO and DF (ie the flax diets) displayed similar horizontal y-axis alignment. The mean center of FSH extended separated itself further into Q1. Lastly, CRN and CTL displayed proximal mean centers located in Q2.

Supplementary Information

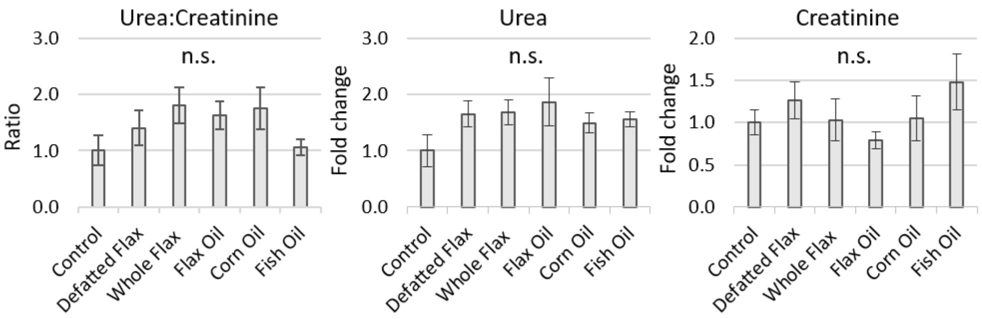

Figure S3. Plasma estimate of renal clearance. Hen blood plasma samples measured via LC-MS/MS. VIP scores of metabolites were analyzed via one-way ANOVA (Duncan's post-test, p<0.05). Groups without a similar letter are significantly different. The sample size from each group is listed in the Materials and Methods section (Section 5.3). Error bars are +/- SEM.

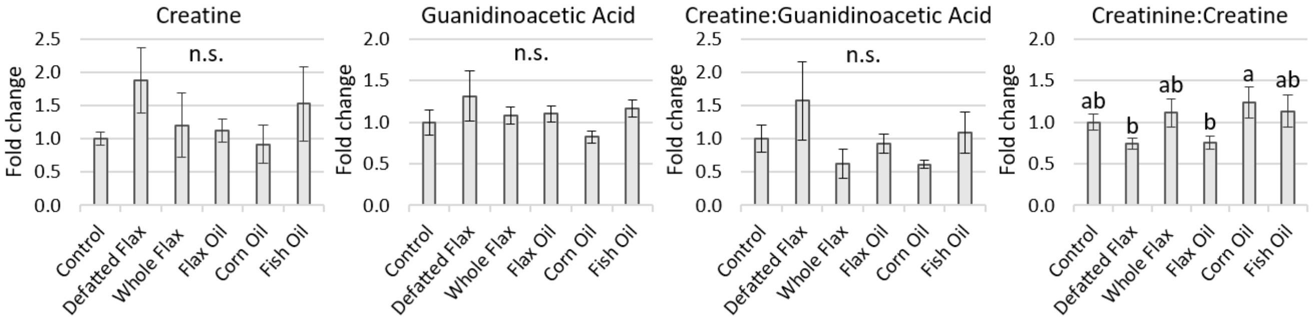

Figure S4. Plasma estimate of creatine metabolism and GAMT activity (i.e. Creatine:Guanidinoacetic Acid). Hen blood plasma samples measured via LC-MS/MS. VIP scores of metabolites were analyzed via one-way ANOVA (Duncan's post-test, p<0.05). Groups without a similar letter are significantly different. The sample size from each group is listed in the Materials and Methods section (Section 5.3). Error bars are +/- SEM.

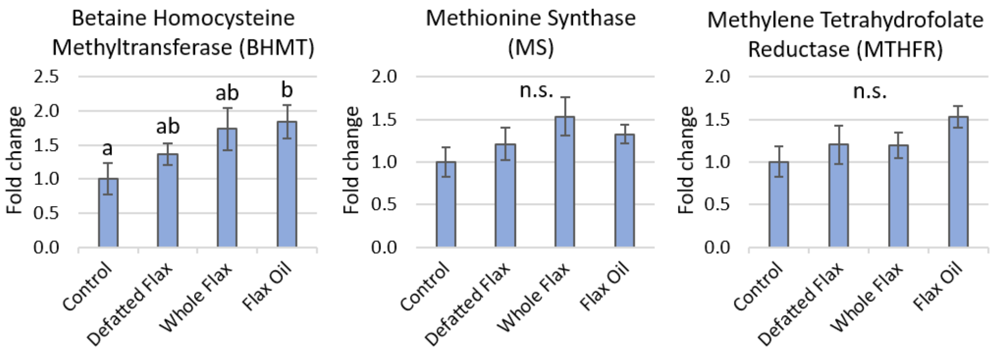

Figure S5. BHMT, MS, and MTHFR gene transcript levels in liver homogenates of hens (using GAPDH has a reference gene). RT-qPCR was conducted on hen liver homogenates to detect the transcript level of genes for the enzymes that regulate Hcy remethylation. Fold changes were analyzed via one-way ANOVA (Duncan's post-test, p<0.05). Groups without a similar letter are significantly different. Error bars are +/- SEM.

Supplementary Information

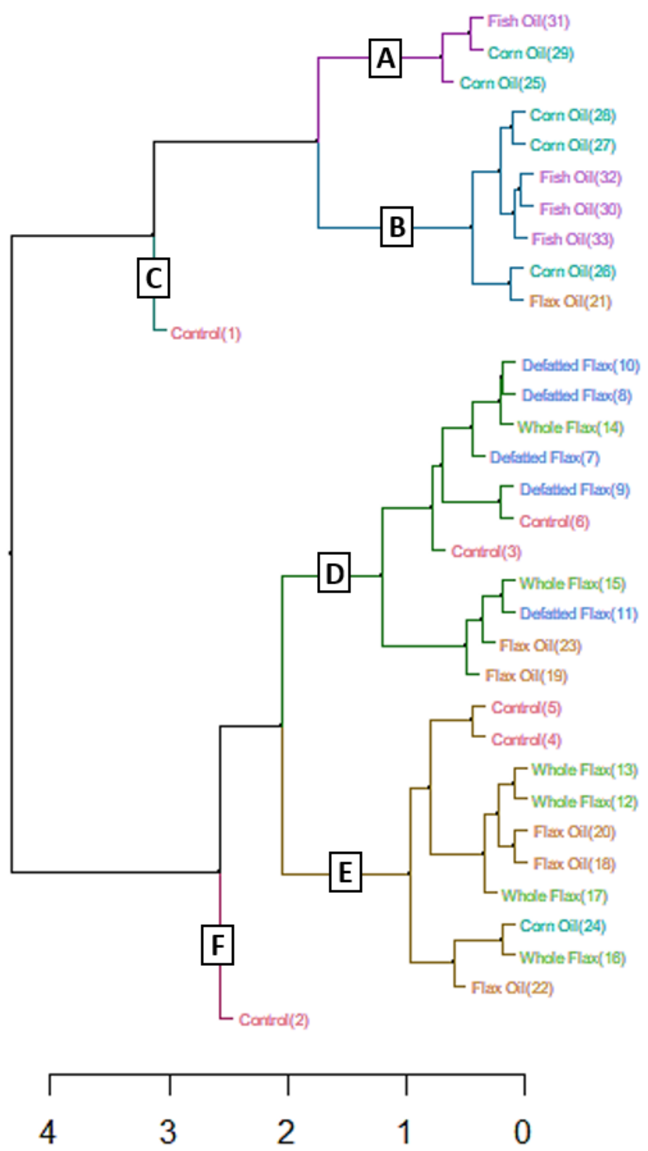

Figure S6. Dendrogram showing K-means clustering of one-carbon donating molecules. K-means clustering analysis was performed on the VIP scores (converted to Z-scores) for "choline, betaine, serine, glycine, DMG, Met:betaine ratio and serine:glycine ratio," in order to determine the hierarchical clustering of blood plasma samples by diet group (k=6 clusters). In order to conduct clustering analysis and dendrogram modeling, each of these metabolites was converted to a standardized Z-score and then evaluated with the R package 'dendextend'. Significantly different clusters are represented by different letters (A-F). All of the FSH samples and five out of six CRN samples grouped into two adjacent clusters (A and B). DFM distinguished itself by grouping within a single cluster (D). Notably, 16 out of 17 samples related to the flaxseed diet (i.e. DFM, WFX or FXO) were grouped into two adjacent clusters (D and E). Overall, WFX samples were mixed with DFM samples and FXO samples. CTL samples expressed high dispersion given that two CTL samples were unique enough to form distinct clusters (C and F). These k-means clusters reflect the results of the PLSDA diagram where we evaluated all 108 metabolites in our dataset (Figure S2), suggesting that the one-carbon donor molecules might be deterministic of large-scale metabolomics, in laying hens or vertebrate animals.

Supplementary Information

Table S1. Plasma levels of molecules that negatively regulate BHMT, in hens by diet group.

BHMT-inhibiting molecule	Plasma Level of Metabolite (by diet group)					
	Control	Defatted Flax	Whole Flax	Flax Oil	Corn Oil	Fish Oil
DMG	moderate	low	low	moderate	moderate	moderate
SAM	moderate	high	moderate	moderate	moderate	moderate
MTA	moderate	high	moderate	moderate	moderate	moderate
Net inhibitory effect	moderate	moderate	low	moderate	moderate	moderate

Table S2. Cox proportional hazard analysis of hen survival, using the corn oil diet as reference.

Diet	exp(coef)[#]	95% c.i.	p-value
Defatted Flax	0.685*	0.471 to 0.997	0.048
Control	0.805	0.567 to 1.143	0.230
Whole Flax	0.825	0.577 to 1.181	0.293
Fish Oil	0.885	0.622 to 1.260	0.500
Flax Oil	0.888	0.625 to 1.263	0.508

[#]An "exp(coef)" below 1.0 indicates reduced risk
*Significantly reduced Cox proportional hazard (p<0.05)

Table S3. F-test results from One-way ANOVAs that were conducted in Figures 3, 4, 5, 6, and 7 (ordered by alphabet).

Parameter	p-value
4-Pyridoxic Acid (Figure 3)	p<0.056
Adenosine (Figure 4)	p<0.05
Adenosine:SAH ratio (Figure 4)	p<0.05
Betaine (Figure 5)	p<0.14
Body Weight of Cancerous Hens (Figure 5)	p>0.30
Body Weight of Normal Hens (Figure 5)	p<0.01
Choline (Figure 4)	p<0.05
Cystathionase (Figure 3)	p<0.05
Cystathionine (Figure 3)	p<0.0001
Cystathionine Beta Synthase (Figure 3)	p<0.05
Cystine (Figure 3)	p>0.30
dAMP (Figure 4)	p<0.05
Dimethylglycine (Figure 6)	p<0.05
Glucuronate (Figure 7)	p<0.01
Glycerophosphrylcholine (Figure 7)	p<0.001
Glycine (Figure 6)	p>0.30
GSSG (Figure 3)	p>0.30
Histidine (Figure 6)	p<0.12
Homocysteic Acid (Figure 3)	p<0.30
Homocysteine (Figure 4)	p>0.30
Homocysteine:SAH (Figure 4)	p>0.066
Met Sulfoxide:Met ratio (Figure 7)	p<0.167
Met:Betaine ratio (Figure 5)	p<0.095
Met:Hcy ratio (Figure 4)	p>0.30
Methionine (Figure 4)	p<0.19
MTA (Figure 4)	p<0.01
Pyridoxamine (Figure 3)	p<0.125
SAH (Figure 4)	p<0.05
SAM (Figure 4)	p<0.05
SAM:Met ratio (Figure 4)	p<0.01
SAM:SAH ratio (Figure 4)	p<0.05
Serine (Figure 6)	p<0.277
Serine:Glycine ratio (Figure 6)	p<0.05
Taurine (Figure 3)	p<0.05
Tryptophan (Figure 6)	p>0.30

Supplementary Information

Supplementary References

1. Osborne, C.B.; Lowe, K.E.; Shane, B. Regulation of folate and one-carbon metabolism in mammalian cells. I. Folate metabolism in Chinese hamster ovary cells expressing Escherichia coli or human folylpoly-gamma-glutamate synthetase activity. *J. Biol. Chem.* **1993**, *268*, 21657–21664.
2. Clare, C.E.; Brassington, A.H.; Kwong, W.Y.; Sinclair, K.D. One-Carbon Metabolism: Linking Nutritional Biochemistry to Epigenetic Programming of Long-Term Development. *Annu. Rev. Anim. Biosci.* **2019**, *7*, 263–287, doi:10.1146/annurev-animal-020518-115206.
3. Millian, N.S.; Garrow, T.A. Human Betaine–Homocysteine Methyltransferase Is a Zinc Metalloenzyme. *Arch. Biochem. Biophys.* **1998**, *356*, 93–98, doi:https://doi.org/10.1006/abbi.1998.0757.
4. Dowhan, W.; Bogdanov, M.B.T.-N.C.B. Chapter 1 Functional roles of lipids in membranes. In *in: Vance D. E. & Vance J. E., Biochemistry of Lipids, Lipoproteins and Membranes, 4th edition*; Elsevier, 2002; Vol. 36, pp. 1–35 ISBN 0167-7306.
5. Åkesson, B. Autoregulation of phospholipid N-methylation by the membrane phosphatidylethanolamine content. *FEBS Lett.* **1978**, *92*, 177–180, doi:10.1016/0014-5793(78)80748-0.
6. Sundler, R.; Akesson, B. Regulation of phospholipid biosynthesis in isolated rat hepatocytes. Effect of different substrates. *J. Biol. Chem.* **1975**, *250*, 3359–3367.
7. Neece, D.J.; Griffiths, M.A.; Garrow, T.A. Isolation and characterization of a mouse betaine-homocysteine S-methyltransferase gene and pseudogene. *Gene* **2000**, *250*, 31–40, doi:https://doi.org/10.1016/S0378-1119(00)00191-8.
8. Finkelstein, J.D. Methionine metabolism in liver diseases. *Am. J. Clin. Nutr.* **2003**, *77*, 1094–1095, doi:10.1093/ajcn/77.5.1094.
9. Finkelstein, J.D. Inborn Errors of Sulfur-Containing Amino Acid Metabolism. *J. Nutr.* **2006**, *136*, 1750S-1754S, doi:10.1093/jn/136.6.1750S.
10. Mudd, S.H.; Brosnan, J.T.; Brosnan, M.E.; Jacobs, R.L.; Stabler, S.P.; Allen, R.H.; Vance, D.E.; Wagner, C. Methyl balance and transmethylation fluxes in humans. *Am. J. Clin. Nutr.* **2007**, *85*, 19–25, doi:10.1093/ajcn/85.1.19.
11. Koutmos, M.; Datta, S.; Pattridge, K.A.; Smith, J.L.; Matthews, R.G. Insights into the reactivation of cobalamin-dependent methionine synthase. *Proc. Natl. Acad. Sci.* **2009**, *106*, 18527 LP – 18532, doi:10.1073/pnas.0906132106.
12. Slow, S.; Garrow, T.A. Liver Choline Dehydrogenase and Kidney Betaine-Homocysteine Methyltransferase Expression Are Not Affected by Methionine or Choline Intake in Growing Rats. *J. Nutr.* **2006**, *136*, 2279–2283, doi:10.1093/jn/136.9.2279.
13. Finkelstein, J.D. Methionine metabolism in mammals. *J. Nutr. Biochem.* **1990**, *1*, 228–237, doi:https://doi.org/10.1016/0955-2863(90)90070-2.
14. Mudd, S.H. and J.R.P. Labile methyl balances for normal humans on various dietary regimens. *Metab. Clin. Exp.* **1975**, *24*, 721–735.
15. Vance, D.E.; Walkey, C.J.; Cui, Z. Phosphatidylethanolamine N-methyltransferase from liver. *Biochim. Biophys. Acta - Lipids Lipid Metab.* **1997**, *1348*, 142–150, doi:https://doi.org/10.1016/S0005-2760(97)00108-2.
16. McMullen, M.H.; Rowling, M.J.; Ozias, M.K.; Schalinske, K.L. Activation and induction of glycine N-methyltransferase by retinoids are tissue- and gender-specific. *Arch. Biochem. Biophys.* **2002**, *401*, 73–80, doi:https://doi.org/10.1016/S0003-9861(02)00030-9.
17. Ye, C.; Sutter, B.M.; Wang, Y.; Kuang, Z.; Tu, B.P. A Metabolic Function for Phospholipid and Histone Methylation. *Mol. Cell* **2017**, *66*, 180-193.e8, doi:10.1016/j.molcel.2017.02.026.
18. Xia, L.; Ma, S.; Zhang, Y.; Wang, T.; Zhou, M.; Wang, Z.; Zhang, J. Daily variation in global and local DNA methylation in mouse livers. *PLoS One* **2015**, *10*, e0118101–e0118101, doi:10.1371/journal.pone.0118101.
19. Xue, C.; Zhao, Y.; Li, L. Advances in RNA cytosine-5 methylation: detection, regulatory mechanisms, biological functions and links to cancer. *Biomark. Res.* **2020**, *8*, 43, doi:10.1186/s40364-020-00225-0.
20. Wang, X.; Lu, Z.; Gomez, A.; Hon, G.C.; Yue, Y.; Han, D.; Fu, Y.; Parisien, M.; Dai, Q.; Jia, G.; et al. N6-methyladenosine-dependent regulation of messenger RNA stability. *Nature* **2014**, *505*, 117–120, doi:10.1038/nature12730.

21. Männistö, P.T.; Kaakkola, S. Catechol-O-methyltransferase (COMT): Biochemistry, Molecular Biology, Pharmacology, and Clinical Efficacy of the New Selective COMT Inhibitors. *Pharmacol. Rev.* **1999**, *51*, 593 LP – 628.
22. Kerr, S.J. Competing Methyltransferase Systems. *J. Biol. Chem.* **1972**, *247*, 4248–4252.
23. Cantoni, G. S-Adenosylamino Acids Thirty Years Later: 1951–1981. In *Biochemistry of S-Adenosylmethionine and Related Compounds*; Palgrave Macmillan: London, 1982; pp. 3–10.
24. Cantoni, G.L.; Chiang, P.K. The Role of S-Adenosylhomocysteine and S-Adenosylhomocysteine Hydrolase in the Control of Biological Methylations. In *Natural Sulfur Compounds*; Cavallini D., Gaull G.E., Z.V. (eds), Ed.; Springer: Boston, MA, 1980; pp. 67–80.
25. Fowler, B. The folate cycle in human disease. *Kidney Int.* **2001**, *59*, S221–S229, doi:10.1046/j.1523-1755.2001.59780221.x.
26. Froese, D.S.; Fowler, B.; Baumgartner, M.R. Vitamin B12, folate, and the methionine remethylation cycle—biochemistry, pathways, and regulation. *J. Inherit. Metab. Dis.* **2019**, *42*, 673–685, doi:10.1002/jimd.12009.
27. Abbasi, I.H.R.; Abbasi, F.; Wang, L.; Abd El Hack, M.E.; Swelum, A.A.; Hao, R.; Yao, J.; Cao, Y. Folate promotes S-adenosyl methionine reactions and the microbial methylation cycle and boosts ruminants production and reproduction. *AMB Express* **2018**, *8*, 65, doi:10.1186/s13568-018-0592-5.
28. Lamers, Y.; Williamson, J.; Ralat, M.; Quinlivan, E.P.; Gilbert, L.R.; Keeling, C.; Stevens, R.D.; Newgard, C.B.; Ueland, P.M.; Meyer, K.; et al. Moderate dietary vitamin B-6 restriction raises plasma glycine and cystathionine concentrations while minimally affecting the rates of glycine turnover and glycine cleavage in healthy men and women. *J. Nutr.* **2009**, *139*, 452–460, doi:10.3945/jn.108.099184.
29. Perry, C.; Yu, S.; Chen, J.; Matharu, K.S.; Stover, P.J. Effect of vitamin B6 availability on serine hydroxymethyltransferase in MCF-7 cells. *Arch. Biochem. Biophys.* **2007**, *462*, 21–27, doi:https://doi.org/10.1016/j.abb.2007.04.005.
30. Wittwer, A.J.; Wagner, C. Identification of the folate-binding proteins of rat liver mitochondria as dimethylglycine dehydrogenase and sarcosine dehydrogenase. Flavoprotein nature and enzymatic properties of the purified proteins. *J. Biol. Chem.* **1981**, *256*, 4109–4115.
31. Kikuchi, G.; Motokawa, Y.; Yoshida, T.; Hiraga, K. Glycine cleavage system: reaction mechanism, physiological significance, and hyperglycinemia. *Proc. Jpn. Acad. Ser. B. Phys. Biol. Sci.* **2008**, *84*, 246–263, doi:10.2183/pjab.84.246.
